# Long non-coding RNA LUCAT1/miR-5582-3p/TCF7L2 axis regulates breast cancer stemness via Wnt/β-catenin pathway

**DOI:** 10.1186/s13046-019-1315-8

**Published:** 2019-07-12

**Authors:** Ang Zheng, Xinyue Song, Lin Zhang, Lin Zhao, Xiaoyun Mao, Minjie Wei, Feng Jin

**Affiliations:** 1 Department of Breast Surgery, the First Affiliated Hospital of China Medical University, No.155 Nanjing Road, Heping Districrt, Shenyang, 110001 People’s Republic of China; 2 Department of Pharmacology, School of Pharmacy, Liaoning Province Key Laboratory of Molecular Targeted Anti-tumor Drug Development and Evaluation, China Medical University, No.77 Puhe Road, Shenbei New District, Shenyang, 110122 People’s Republic of China; 3 Department of Surgery, Hwamei Hospital, University of Chinese Academy of Sciences, (Ningbo No.2 Hospital). No.41 Xibei Road, Haishu District, NingBo, 315000 People’s Republic of China

**Keywords:** Breast cancer, Stemness, LUCAT1, miR-5582-3p, Wnt/β-catenin signaling pathway

## Abstract

**Background:**

The mechanism underlying breast cancer stem cell (BCSCs) characteristics remains to be fully elucidated. Accumulating evidence implies that long noncoding RNAs (lncRNAs) play a pivotal role in regulating BCSCs stemness.

**Methods:**

LncRNA LUCAT1 expression was assessed in breast cancer tissues (*n* = 151 cases) by in situ hybridization. Sphere-formation assay and colony formation assay were used to detect cell self-renewal and proliferation, respectively. RNA immunoprecipitation, RNA pull down and luciferase reporter assays were used to identify LUCAT1 and TCF7L2 as the direct target of miR-5582-3p. The activity of the Wnt/β-catenin pathway was analyzed by TOP/FOP-Flash reporter assays, western blot and immunohistochemistry (IHC).

**Results:**

This study found LUCAT1 expression was related to tumor size (*p* = 0.015), lymph node metastasis (*p* = 0.002) and TNM staging (*p* < 0.001). High LUCAT1 expression indicated a shorter overall survival (*p* = 0.006) and disease-free survival (*p* = 0.011). Furthermore, LUCAT1 was more expressed in BCSCs than in breast cancer cells (BCCs) by lncRNA microarray chips. LUCAT1 up-regulation promoted proliferation of BCCs, while LUCAT1 down-regulation inhibited self-renewal of BCSCs. MiR-5582-3p was directly bound to LUCAT1 and TCF7L2 and negatively regulated their expression. LUCAT1 affected Wnt/β-catenin pathway.

**Conclusions:**

LUCAT1 might be a significant biomarker to evaluate prognosis in breast cancer. LUCAT1 increased stem-like properties of BCCs and stemness of BCSCs by competitively binding miR-5582-3p with TCF7L2 and enhancing the Wnt/β-catenin pathway. The LUCAT1/miR-5582-3p/TCF7L2 axis provides insights for regulatory mechanism of stemness, and new strategies for clinical practice.

**Electronic supplementary material:**

The online version of this article (10.1186/s13046-019-1315-8) contains supplementary material, which is available to authorized users.

## Background

On a global scale, breast cancer is the most frequent malignancy and the leading cause of cancer death in women, with 24.2% of all cancers and 15% of all cancer death in 2018 [1, 2]. In China, breast cancer accounts for 12.2% of new cases and 9.6% of cancer deaths [3]. In the field of therapy, many clinicians are striving for the goal, to identify areas where optimal care may be achieved with ‘escalating’ or ‘de-escalating’ treatment. As ‘de-escalation’ requires more valuable evidence and rigorous judgment, filtering novel biomarkers with prognostic function is considered to be an effective way.

Heterogeneous clusters of tumor cells exist in solid tumors, a special subgroup of which is called cancer stem cells (CSCs), characterized by self-renewal and pluripotency [4, 5]. Breast cancer stem cells (BCSCs) are regarded as the source of tumor development, differentiation, invasion and metastasis, drug resistance and recurrence in breast cancer [6–8]. Stem cells has shown attractive prospects in therapy. Therefore, studies on stemness regulation of BCSCs are significant in theory and clinical practice.

Long non-coding RNAs (lncRNAs) are a series of transcript RNA longer than 200 nucleotides without potentials of protein-coding [9]. LncRNAs recruit transcription factors to regulate gene expression, or interact with microRNAs (miRNAs) and influence the stability of mRNAs [10]. LncRNAs participate in epigenetic regulation in pathophysiology [11, 12]. LncRNAs are involved in the tumorigenesis and progression and considered to be a potential biomarker for cancer diagnosis, prognosis and therapy [13] .

In the present study, large scale screening for the differentially expressed lncRNAs between BCCs and BCSCs, is performed by lncRNAs microarray. After filtration by bioinformatics analysis and consultation based on previous studies, LUCAT1(lung cancer associated transcript 1) is selected as the target. LUCAT1 is located at 5q14.3, firstly found in the airway epithelium of smokers [13, 14]. LUCAT1 is significantly more-expressed in non-small cell lung cancer (NSCLC) tissues than normal tissues. Its high expression is correlated with high TNM staging, positive lymph node metastasis and poor prognosis [15]. LUCAT1 is related to cisplatin resistance in ovarian cancer and tumorigenesis in colorectal cancer [16]. Nonetheless, little is known regarding the expression of LUCAT1 in breast cancer and the stemness regulation of LUCAT1 in BCSCs.

The competing endogenous RNA (ceRNA) hypothesis is a classical mode of gene expression regulation. LncRNAs exert a ‘miRNA Sponge’ by competitively binding miRNAs to antagonize the function of miRNAs in inhibiting specific target mRNAs [17, 18]. Several studies reported that lncRNAs, as ceRNA, affected tumorigenesis and progression, reflecting a new level of post-transcriptional regulation of genes and providing new insights for the molecular mechanism of CSCs [19, 20].

Herein, we assessed LUCAT1 expression in 151 breast cancer specimens and firstly reported the association of LUCAT1 with clinical pathology factors and prognosis in breast cancer. Based on big data, the relationship between LUCAT1 and stemness marker was analyzed. LUCAT1 functioned as a miR-5582-3p ‘sponge’ and targeted TCF7L2 (transcription factor 7-like 2), mediated Wnt/β-catenin pathway, and regulated self-renewal of BCSCs and proliferation of BCCs, in vitro *and* in vivo. The LUCAT1/miR-5582-3p/TCF7L2 axis might provide theoretical support for finding new diagnostic markers and therapeutic targets of breast cancer.

## Methods

### Data extraction and TCGA analysis

Gene expression was downloaded from TCGA-BRCA (https://cancergenome.nih.gov/). The edgeR package was used to normalize gene expression. Differential expressions of LUCAT1 in CD44^+^CD24^−^ (BCSCs phenotype marker) and non CD44^+^CD24^−^ patients were analyzed [21]. Expressions of OCT4, ABCC1, Wnt1, HIF-1α in LUCAT1-high and LUCAT1-low patients were analyzed.

### Patient specimens

All patients were diagnosed with infiltrative ductal carcinoma in the First Affiliated Hospital of China Medical University. Prior to operation, patients did not receive chemo- or radiotherapy. Breast cancer specimens (*n* = 151 cases) were obtained from patients hospitalized from September 2008 to December 2009. The follow-up information was collected from patients or immediate family members through telephone twice a year. Fresh cancer tissues and the matched adjacent normal tissues (*n* = 26 pairs) were obtained from patients hospitalized from June to July 2018, without follow-up information. Fresh tissues were snap frozen in liquid nitrogen immediately after surgery and stored. This study was approved by Ethics Committee of China Medical University (Approval number: AF-SOP- 07-1.1-01).

### In situ hybridization (ISH)

Slides were processed by 1 mL/L DEPC-treated water and APES glue. All liquid and experimental apparatus were treated to remove RNA enzymes. Firstly, the sections were de-waxed by xylene and rehydrated in graded alcohol series. Next, 3% hydrogen peroxide was used to block endogenous peroxidase activity and 3% fresh citric acid diluted pepsin was used to expose mRNA. Then, slides were incubated at 37 °C for 2 h with 20 μL preliminary hybrid liquid, followed by an overnight incubation with 20 μL digoxin-labeled oligonucleotide probe and hybrid liquid at 37 °C. The following step was to add blocking solution, biotinylated rat anti digoxin and SABC, according to protocol in LncRNAs ISH Kit (Boster). DAB staining was evaluated by two pathologists who were blinded to the experiment separately. LUCAT1 expression was estimated by double score semi-quantitative analysis, as previously described [22]. Patients were categorized into two groups: LUCAT1-high (score > 3) and LUCAT1-low (score ≤ 3). The probe used for LUCAT1 detection was shown in Additional file 1: Table S1.

### Cell lines and culture

Normal breast epithelial cell line MCF-10A, breast cancer cell lines MCF-7 and T47D were obtained from ATCC (Manassas, VA, USA). MCF-10A, MCF-7 and T47D cell lines were cultured in high-glucose (4.5 mg/ml) DMEM with 10% (*v*/v) FBS (HyClone). MCF-7 CSCs and T47D CSCs were induced and cultured in DMEM-F12 (Gibco, Thermo Fisher Scientific), containing 2% B27 (Gibco), b-FGF 10 μg/L (Promega), EGF 20 μg/L (Promega), as previously described [23]. All cells were maintained at 37 °C in a 5% CO_2_ and 95% air incubator.

### Cell transfection and virus infection

MiR-5582 mimic and inhibitor (RIOBOBIO, Guangzhou, China) were transfected into MCF-7 and T47D by Lipofectamine 3000 (Invitrogen), in accordance with protocol. For shRNA knock-down analysis, lentiviral vectors (GV248) were purchased from Genechem Co., Ltd. (Shanghai, China). MCF-7 CSCs and T47D CSCs were transfected with sh-Ctrl or sh-LUCAT1 lentiviral transduction particles (MOI = 20) with 5 μg/mL polybrene (Genechem). For cDNA knock-in analysis, lentiviral vectors (GV502) were purchased from Genechem. MCF7 and T47D were transfected with NC-cDNA or LUCAT1-cDNA (MOI = 20) with polybrene. The medium was replaced with fresh culture medium 24 h after transfection. Stably transfected cells were selected by puromycin (1 μg/ml). The selection was repeated 2–3 times till green fluorescent protein (GFP) was observed in all cells under a fluorescence microscope (Nikon TE 2000-U, Japan). The target siRNAs against LUCAT1 for RNAi, constitution of the vector system were shown in Additional file 1: Table S1.

### Quantitative real-time polymerase chain reaction (qRT-PCR)

Trizol reagent kit (CWBIO, China) was used to extract total RNA. RNA concentration was detected by NanoDrop 2000 spectrophotometer (Termo Scientific, USA). cDNA was compounded using the PrimeScript™ RT reagent Kit with gDNA Eraser (Takara, Japan). The miRNA qRT-PCR Starter kit (RIOBOBIO) was used for reverse transcription of miRNA. The primers of U6 and miR-5582 were purchased from RIOBOBIO. SYBR® Green Realtime PCR Msater Mix (TOYOBO, Japan) was used. With 2^-ΔΔCT^ method (β-actin as the reference), concentration error could be eliminated. The primer sequences were presented in Additional file 1: Table S1.

### RNA isolation of nuclear and cytoplasmic fractions

The NE-PER™ Nuclear and Cytoplasmic Extraction Reagents Kit (Thermo Scientific, USA) was applied to isolate and collect cytosolic and nuclear fractions. The experiment generally followed manufacturer’s protocol. RNA levels of LUCAT1, U6 (nuclear control transcript), and GAPDH (cytoplasmic control transcript) were analyzed by qRT-PCR.

### Western blot

Cells or tissues were washed with cold PBS and lysed in RIPA buffer containing 1% proteinase inhibitor cocktail solution (Sigma-Aldrich) on ice for 30 min. The NE-PER™ Nuclear and Cytoplasmic Extraction Reagents Kit (Thermo Scientific, USA) was applied to isolate nuclear fractions. Lysates were centrifuged, and protein was quantified using the BCA assay kit (Beyotime, Jiangsu, China). SDS-PAGE electrophoresis was performed on proteins from lysates and transferred them to PVDF membranes (Millipore, Bedford, MA). Membranes were incubated with primary antibody overnight at 4 °C, and then detected by Enhanced Chemiluminescence Detection Kit (BOSTER, USA). Lamin B1 was used as an endogenous control of cell nuclear fraction. All antibodies used were shown in Additional file 2: Table S2.

### Immunohistochemistry (IHC)

IHC was performed as previously described [22] . Ultra-sensitive™ S-P Kit (Maixin-Bio, China) was used. Briefly, sections from paraffin-embedded tumor tissues from transplanted nude mice were incubated with primary antibodies. Results were evaluated by two pathologists who were blinded to the experiment separately.

### Flow cytometry assay

All cell lines were digested with 0.25% trypsin and washed with PBS three times. Then cells were resuspended in 100 μL PBS and incubated with anti-CD44-APC (1.25 μl/ test) and anti-CD24-PE (5 μl/ test) (Bio legend, San Diego, USA), or with their controls at 4 °C for 30 min. After incubation, the cells were washed two times with PBS and suspended in 300 μL PBS. Analysis was performed on a MACSQuantTM Flow Cytometer (Miltenyi Biotec).

### Sphere-formation assay and colony formation assay

Sphere-formation assay was performed as previously described [24]. Briefly, MCF-7 CSCs and T47D CSCs suspension (1 × 10^3^/well) was plated in ultra-low adhesion plates (Corning, Kraemer, CA). The cells grown in 2 ml serum-free DMEM-F12 with 10 μg/L bFGF, 20 μg/L EGF and 2% B27. For Colony formation assay, 2 × 10^3^ MCF-7 and T47D cells were seeded into 6 cm petri dishes and cultured at 37 °C in 5% CO_2_. After 14 days, cells were washed with PBS and fixed in paraformaldehyde for 15 min, stained with 0.5% crystal violet for 15 min.

### Luciferase reporter and TOP/FOP-flash reporter assays

Full-length LUCAT1 sequence of wild-type (WT) and mutant-type (MUT) miRNA binding site was constructed in Genechem Co., Ltd. (Shanghai, China). LUCAT1 WT/MUT were transfected into MCF-7 with miR-5582-3p mimic/NC mimic. Similarly, the binding sites for miR-5582-3p in the 3′-untranslated region (3′-UTR) sequence of TCF7L2 were obtained from Genechem. TCF7L2 3′-UTR WT/MUT were transfected into MCF-7 with miR-5582-3p mimic/NC mimic. The Dual-Luciferase Reporter Assay (Promega) was applied according to the manufacturer’s instructions. For the TOP/FOP-Flash assay, TOP/FOP-Flash (Genechem) was co-transfected into cells along with LUCAT1 silence or overexpression vector, miR-5582-5p mimic or inhibitor, and/or the miRNA control. The TOP/FOP-Flash values were normalized to the *Renilla reniformis* (Promega) reading and the TOP/FOP ratio was measured, as previously described [25]. Experiments were performed in triplicate.

### RNA immunoprecipitation (RIP)

The Magna RIP™ RNA-Binding Protein Immunoprecipitation Kit (Millipore, Bedford, MA, USA) was applied according to the manufacturer’s protocol [26]. MCF7 cells were transfected with NC-cDNA or LUCAT1-cDNA. Complete RIP lysis buffer was used to lyse cells. Magnetic beads conjugated with anti-Argonaute 2 (AGO2) (Millipore) or control anti-immunoglobulin G (IgG) antibody were used to incubate the cell extract. The cell extract was incubated for 6 h at 4 °C. As the protein beads were removed, qRT-PCR was conducted for the purification of RNA.

### RNA pull-down assay

The pull-down assay was performed as previously described [26]. Briefly, purified biotinylated LUCAT1 and LUCAT1-mut transcripts were purchased from Sangon Biotech (Shanghai, China). One milligram of MCF-7 cell lysates was incubated with three micrograms of purified biotinylated transcripts for 1 h at 25 °C. The biotin-coupled RNA complexes were isolated by streptavidin agarose beads (Invitrogen) and microRNAs abundance in the pull-down material was analyzed by qRT-PCR.

### Xenograft model

1.0 × 10^6^ of MCF-7 cells stably transfected with NC-cDNA and LUCAT1-cDNA were suspended in 100 μl PBS and injected into each mammary fat pad of 3/4-week-old female BALB/c (nu/nu) mice (Hua Fukang Biological Technologies Inc., Beijing). The mice were randomized into four groups (*n* = 3 per group). Once the tumors had formed, we injected 1.5 nmol miR-5582-3p agomir or agomir NC (RIOBOBIO) at multiple points in tumors, at an interval of 2 days between each injection (i.e., day 10, 13, 16 …) [26] . The tumor diameter and weights were measured every other day. The tumors were removed and weighed until the largest tumor was close to 2 cm. Tumor volume (mm^3^) was measured by a digital caliper and calculated as (width) ^2^ × (length/2). All mice were bred at pathogen-free conditions in the Animal Center. All animal experiments were approved by the Institutional Animal Care and Use Committee of China Medical University (Approval number: 2017007 M).

### Statistical analysis

Statistical analyses were conducted in GraphPad Prism 6.0 (La Jolla, CA, USA) and SPSS 24.0 (Chicago, IL, USA). Results were presented as the mean ± standard deviation for at least three experiments. Student’s independent t test was used between two groups. The relationship between LUCAT1 and clinical pathology factors was examined by Pearson chi-square tests, Fisher’s exact tests and logistic regression analyses. Survival probabilities were judged by the Kaplan-Meier and assessed by a log-rank test. Probability values less than 0.05 were considered statistically significant.

## Results

### LUCAT1 is over-expressed in the BCSCs than BCCs and related to breast cancer stemness

Since the induction and culture of BCSCs was matured in our research group [23], the present study briefly verified the results. The morphology of MCF-7 and T47D changed obviously and size of mammospheres rapidly increased 7–8 days later (Additional file 3: Figure S1a). MCF-7 CSCs and T47D CSCs possessed typical properties, such as high CD44^+^CD24^−^ phenotype, high expression of CSCs markers (OCT4, SOX2 and Nanog) in mRNA and protein levels (Additional file 3: Figure S1b-d).

**Fig. 1:**
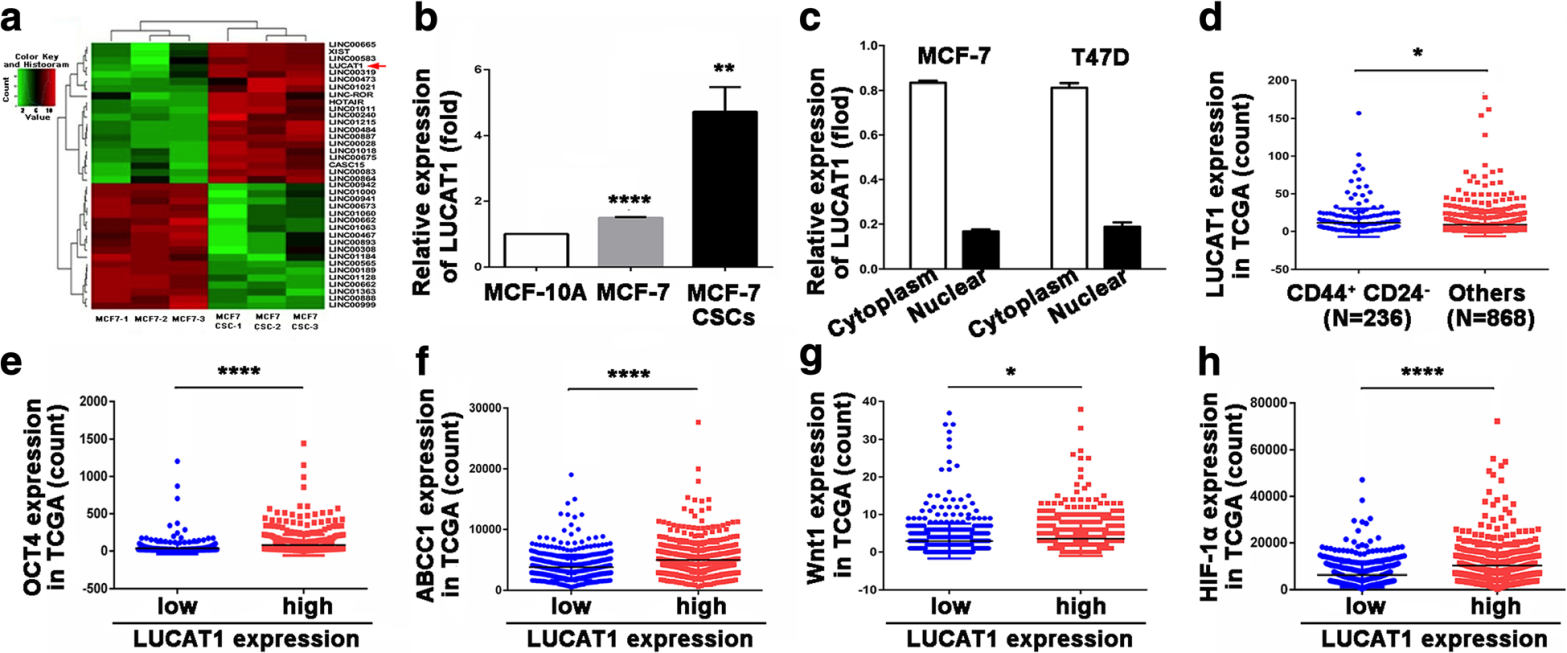
LUCAT1 is over-expressed in the BCSCs compared to BCCs and related to BC stemness. **a** Heat maps of differential lncRNAs between MCF-7 CSCs and MCF-7 in microarray expression profile. **b** mRNA expression level of LUCAT1 in MCF-10A, MCF-7 and MCF-7 CSCs detected by qRT-PCR. **c** RT-qPCR was performed to measure the relative expression of LUCAT1 in nuclear and cytoplasm. The majority of LUCAT1 was located in the cytoplasm according to the nuclear mass separation assay. **d** mRNA expression level of LUCAT1 in patients with CD44 + CD24- phenotype and others from RNA sequencing data of TCGA database. **e** mRNA expression level of OCT4 in LUCAT1-high and LUCAT1-low patients from TCGA database. **f** mRNA expression level of ABCC1 in LUCAT1-high and LUCAT1-low patients from TCGA database. **g** mRNA expression level of Wnt1 in LUCAT1-high and LUCAT1-low patients from TCGA database. **h** mRNA expression level of HIF-1α in LUCAT1-high and LUCAT1-low patients from TCGA database. *P < 0.05, **P < 0.01, ***P < 0.001, ****P < 0.0001

To investigate differentially expressed lncRNAs between BCCs and BCSCs, we performed lncRNA microarray chips (Oebiotech, Shanghai, China). LUCAT1 expression in MCF-7 CSCs was significantly higher than that in MCF-7 cells (Fig. 1a and Additional file 4: Table S3). We used qRT-PCR to confirm the result of microarray profiling (*p* = 0.0017). Meanwhile, LUCAT1 expression in MCF-7 cells was higher than that in MCF-10A cells (*P* < 0.0001) (Fig. 1b). The subcellular localization of LUCAT1 in MCF-7 cell lines was determined using the nuclear mass separation assay, and it was found that LUCAT1 was mainly located in the cytoplasm (Fig. 1c). We detected LUCAT1 expression in 26 pairs of fresh specimens by qRT-PCR and found LUCAT1 expression in cancer tissues was higher than matched adjacent normal tissues (*p* < 0.01, Additional file 5: Figure S2a).

To make a preliminary study on the association of LUCAT1 with breast cancer stemness, we used the TCGA RNA Seq data. LUCAT1 expression was distinctly higher in patients with CD44^+^CD24^−^ phenotype (*n* = 236) than others (*n* = 868) (*p* = 0.0274, Fig. 1d). The stemness markers including OCT4 (*p* < 0.0001), ABCC1 (a CSCs chemo-resistance gene, *p* < 0.0001), Wnt1 (a CSCs self-renewal related gene, *p* = 0.0209) and HIF-1α (an important factor in promoting CSCs status, *p* < 0.0001) expressed higher in LUCAT1-high patients than LUCAT1-low patients (Fig. 1e-h) [27]. In 26 fresh specimens, LUCAT1 mRNA expression (qRT-PCR) was positively correlated with SOX2 protein expression (IHC) (r = 0.6701, *p* < 0.001, Additional file 5: Figure S2b). Furthermore, there was also a positive correlation between LUCAT1 mRNA expression (ISH) and SOX2 protein expression (IHC) in 151 samples (Table 3). The results added to growing evidence that LUCAT1 was associated with oncogenic properties and increased stem phenotype.

### Association of LUCAT1 with clinical pathology factors and prognosis

**Fig. 2:**
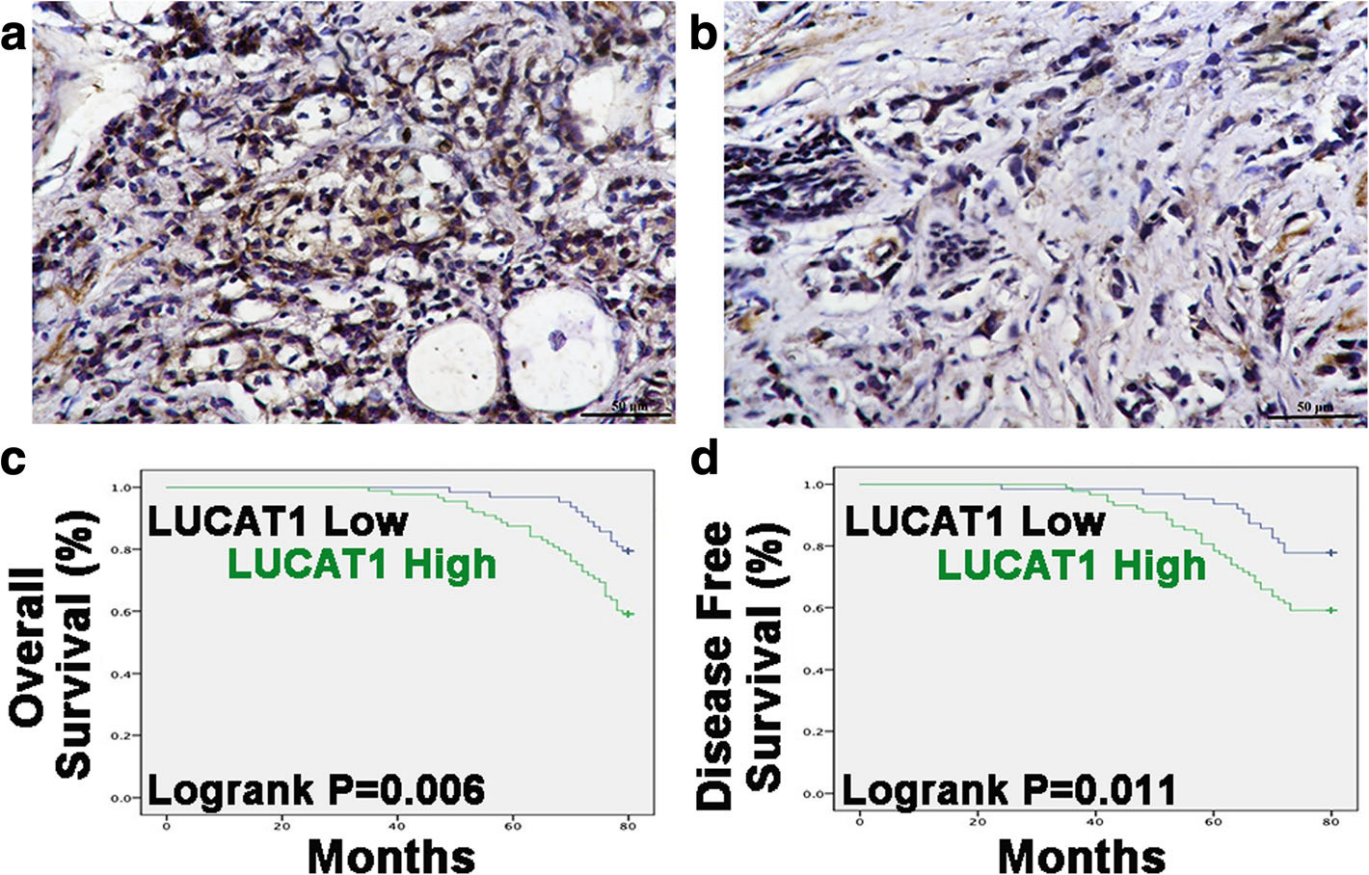
ISH results and prognostic significance of LUCAT1. **a** High expression of LUCAT1 in ISH. **b** Low expression of LUCAT1 in ISH. **c** Kaplan-Meier survival analysis of OS based on LUCAT1 expression in our cohort (*p* = 0.006). **d** Kaplan-Meier survival analysis of DFS based on LUCAT1 expression in our cohort (*p* = 0.011). ISH Original magnification, × 400. Scale bars, 50 μm

To elucidate whether LUCAT1 contributed to breast cancer, we evaluated the expression levels of LUCAT1 in 151 breast cancer samples by ISH. High and low expression were shown in Fig. 2 a-b. To further elucidate how LUCAT1 was involved in the breast cancer development, we analyzed the correlation of LUCAT1 expression with clinical pathology factors. There was a significant correlation between LUCAT1 expression and tumor size (*p* = 0.015), lymph node metastasis (*p* = 0.002) and TNM staging (*p* < 0.001) (Table 1). TNM staging was an independent factor of LUCAT1 expression (Table 2). To assess the association of LUCAT1 expression with OS and DFS, we performed Kaplan-Meier survival analysis. LUCAT1-high patients had significantly lower OS (*p* = 0.006) and DFS (*p* = 0.011) than LUCAT1-low patients (Fig. 2e-f). Therefore, LUCAT1 could be a significant biomarker to evaluate prognosis of breast cancer patients.

**Table 1:** Univariate analysis of LUCAT1 and clinical pathology factors

Factors	Number (%)	LUCAT1 expression		χ^2^	*P-value*	Crude OR (95% CI)
		High (%)	Low (%)			
Age (years)				0.872	0.707^a^	
< 40	25 (16.6)	15 (40.0)	10 (60.0)		0.778^b^	0.857 (0.294–2.497)
41–50	53 (35.1)	29 (59.6)	24 (45.3)		0.416^b^	0.690 (0.283–1.685)
51–60	40 (26.5)	23 (57.5)	17 (42.5)		0.594^b^	0.773 (0.300–1.992)
> 61	33 (21.9)	21 (63.6)	12 (36.4)			Reference
Tumor size (cm)				5.94	0.015^a^	
≥ 3	87 (57.6)	58 (66.7)	29 (33.3)		0.016^b^	2.267 (1.168–4.399)
< 3	64 (42.4)	30 (46.9)	34 (53.1)			Reference
LN Metastases				9.239	0.002^a^	
negative	86 (57.0)	41 (47.7)	45 (52.3)			Reference
positive	65 (43.0)	47 (72.3)	18 (27.7)		0.003^b^	2.866 (1.439–5.706)
ER				0.374	0.541^a^	
positive	58 (38.4)	32 (55.2)	26 (44.8)			Reference
negative	93 (61.6)	56 (60.2)	37 (39.8)		0.541^b^	1.230 (0.633–2.388)
PR				2.199	0.138^a^	
positive	59 (39.1)	30 (50.8)	29 (49.2)			Reference
negative	92 (60.9)	58 (63.0)	34 (37.0)		0.139^b^	1.649 (0.850–3.200)
Her2				1.007	0.316^a^	
positive	101 (66.9)	56 (55.4)	45 (44.6)			Reference
negative	50 (33.1)	32 (64.0)	18 (36.0)		0.317^b^	1.429 (0.711–2.871)
Histological grade				5.930	0.052^a^	
2	124 (82.1)	77 (62.1)	47 (37.9)			Reference
3	14 (9.3)	4 (28.6)	10 (71.4)		0.023^b^	0.244 (0.072–0.823)
unrated	13 (8.6)	7 (53.8)	6 (46.2)		0.563^b^	0.712 (0.226–2.247)
Molecular typing				2.332	0.506^a^	
Luminal A	60 (39.7)	33 (55.0)	27 (45.0)		0.678^b^	1.222 (0.474–3.154)
Luminal B	48 (31.8)	32 (66.7)	16 (33.3)		0.174^b^	2.000 (0.736–5.438)
Her-2	19 (12.6)	11 (57.9)	8 (42.1)		0.607^b^	1.375 (0.409–4.622)
Basal-like	24 (15.9)	12 (50.0)	12 (50.0)			Reference
TNM staging				18.105	< 0.001^a^	
I	38 (25.2)	12 (31.6)	26 (68.4)			Reference
II	75 (49.6)	46 (61.3)	29 (38.7)		0.003^b^	3.437 (1.503–7.858)
III	38 (25.2)	30 (78.9)	8 (21.1)		< 0.001^b^	8.125 (2.879–22.927)

P ^a^: Pearson chi-square tests or Fisher’s Exact Test. P ^b^: logistic regression analyses

**Table 2:** Multivariate analysis of LUCAT1 and clinical pathology factors

Factors	*P-value*	Adjusted OR (95% CI)
TNM staging		
I		Reference
II	0.007	3.460 (1.405–8.518)
III	0.002	6.310 (1.952–20.398)
Tumor size (cm)		
≥ 3	0.491	1.434 (0.514–3.998)
< 3		Reference
LN Metastases		
negative		Reference
positive	0.134	2.197 (0.785–6.150)

### LUCAT1 contributes to stem-like properties of BCCs and stemness of BCSCs

**Fig. 3:**
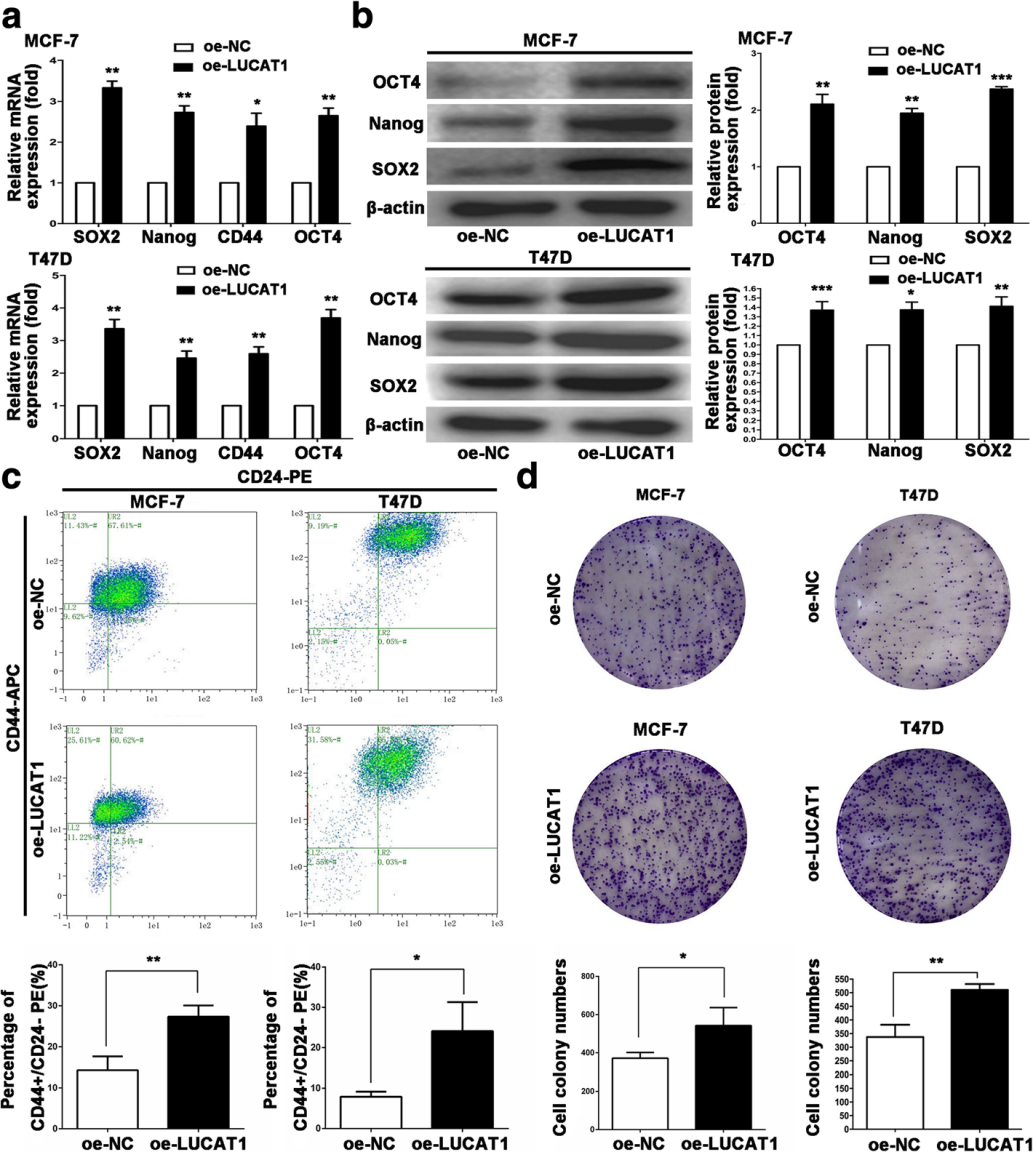
LUCAT1 enhances stem-like properties of BCCs. **a** Expression of stemness markers (SOX2, Nanog, CD44 and OCT4) was detected by qRT-PCR in stable LUCAT1-cDNA transfected MCF-7 and T47D cells. **b** Expression of stemness markers (OCT4, Nanog and SOX2) was detected in LUCAT1-overexpressing MCF-7 and T47D cells by WB. **c** Rate of the CD44 + CD24- cells was detected in stable LUCAT1-cDNA transfected MCF-7 and T47D cells by flow cytometry. **d** Proliferation ability was analyzed in LUCAT1-overexpressing MCF-7 and T47D cells by colony formation assay. Data are presented as the mean ± SD of three independent experiments performed in triplicate. *P < 0.05, **P < 0.01, ***P < 0.001, ****P < 0.0001

To investigate whether LUCAT1 influenced stem-like properties of BCCs, we constructed LUCAT1-overexpressing (oe-LUCAT1) MCF-7 and T47D cells (Additional file 5: Figure S2c). SOX2, Nanog, OCT4 expression and the rate of CD44^+^CD24^−^ cells were upregulated in oe-LUCAT1 MCF-7 and T47D cells in mRNA and protein levels (Fig. 3a-c). We detected cell proliferation ability by colony formation assay. Compared with oe-NC group, oe-LUCAT1 cells formed more and bigger colonies (Fig. 3d).

**Fig. 4:**
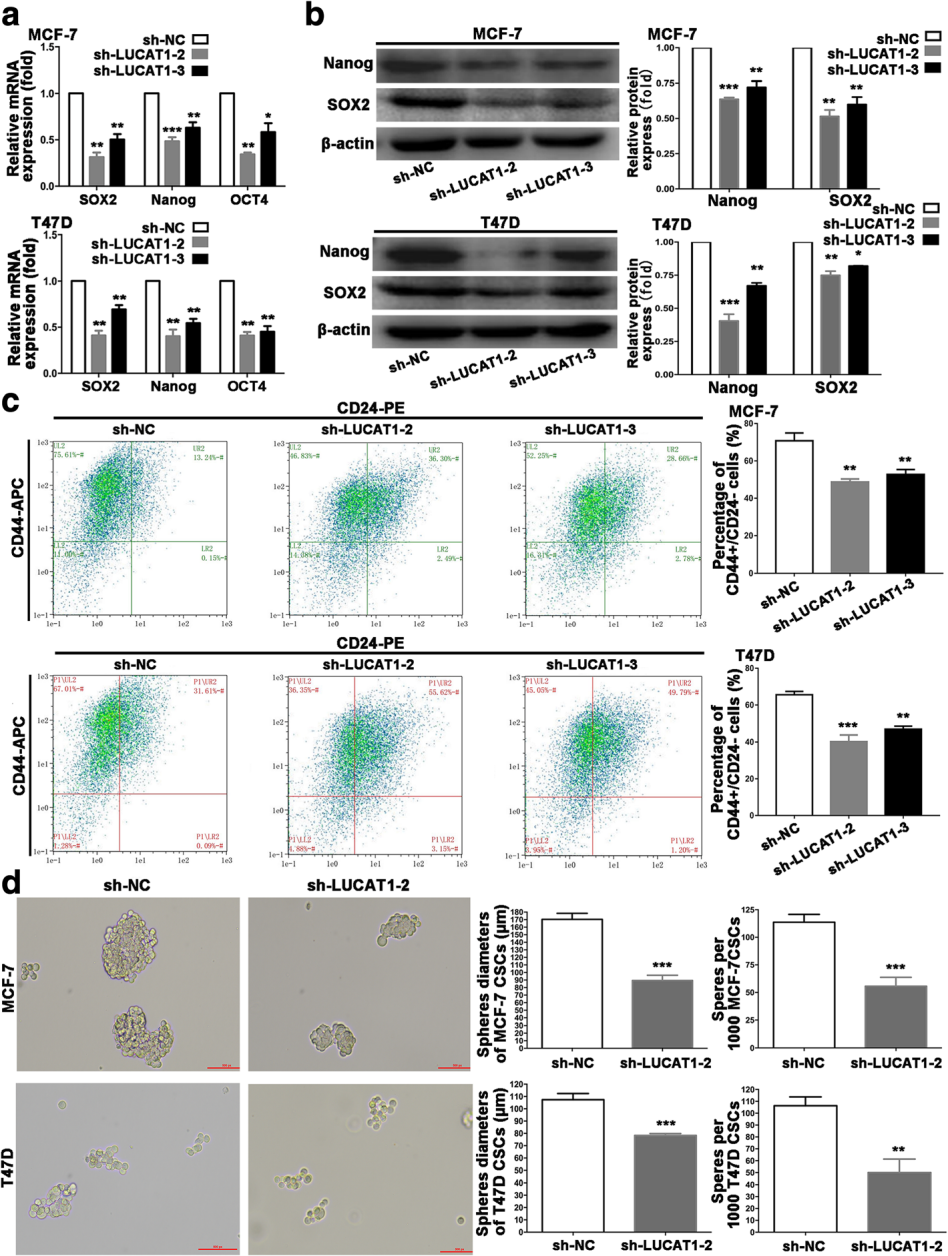
LUCAT1 contributes to stemness of BCSCs. **a** Expression of stemness markers (SOX2, Nanog and OCT4) was detected by qRT-PCR in stable sh-LUCAT1 transfected MCF-7 CSCs and T47D CSCs. **b** Expression of stemness markers (Nanog and SOX2) was detected in LUCAT1-silencing MCF-7 CSCs and T47D CSCs by WB. **c** Rate of the CD44 + CD24− cells in MCF-7 CSCs and T47D CSCs transfected with sh-LUCAT1 subpopulation by flow cytometry. Original magnification, × 400. Scale bars, 50 μm. **d** Self-renewal ability was detected in LUCAT1-silencing MCF-7 CSCs and T47D CSCs by sphere formation assays. Data are presented as the mean ± SD of three independent experiments performed in triplicate. *P < 0.05, **P < 0.01, ***P < 0.001, ****P < 0.0001

To illustrate whether LUCAT1 impacted stem phenotype of BCSCs, we established stable LUCAT1-silencing (sh-LUCAT1) MCF-7 CSCs and T47D CSCs (Additional file 5: Figure S2d). SOX2, Nanog, OCT4 expression and rate of CD44^+^CD24^−^ cells were down-regulated in sh-LUCAT1 MCF-7 CSCs and T47D CSCs (Fig. 4a-c). We evaluated CSCs self-renewal ability by sphere-formation assay and found LUCAT1 silencing reduced mammosphere diameter and quantity (Fig. 4d). All these results suggested that LUCAT1 contributes to stem-like properties of BCCs and stemness of BCSCs.

### LUCAT1 competitively binds miR-5582-3p with TCF7L2 and enhances Wnt/β-catenin pathway

**Fig. 5:**
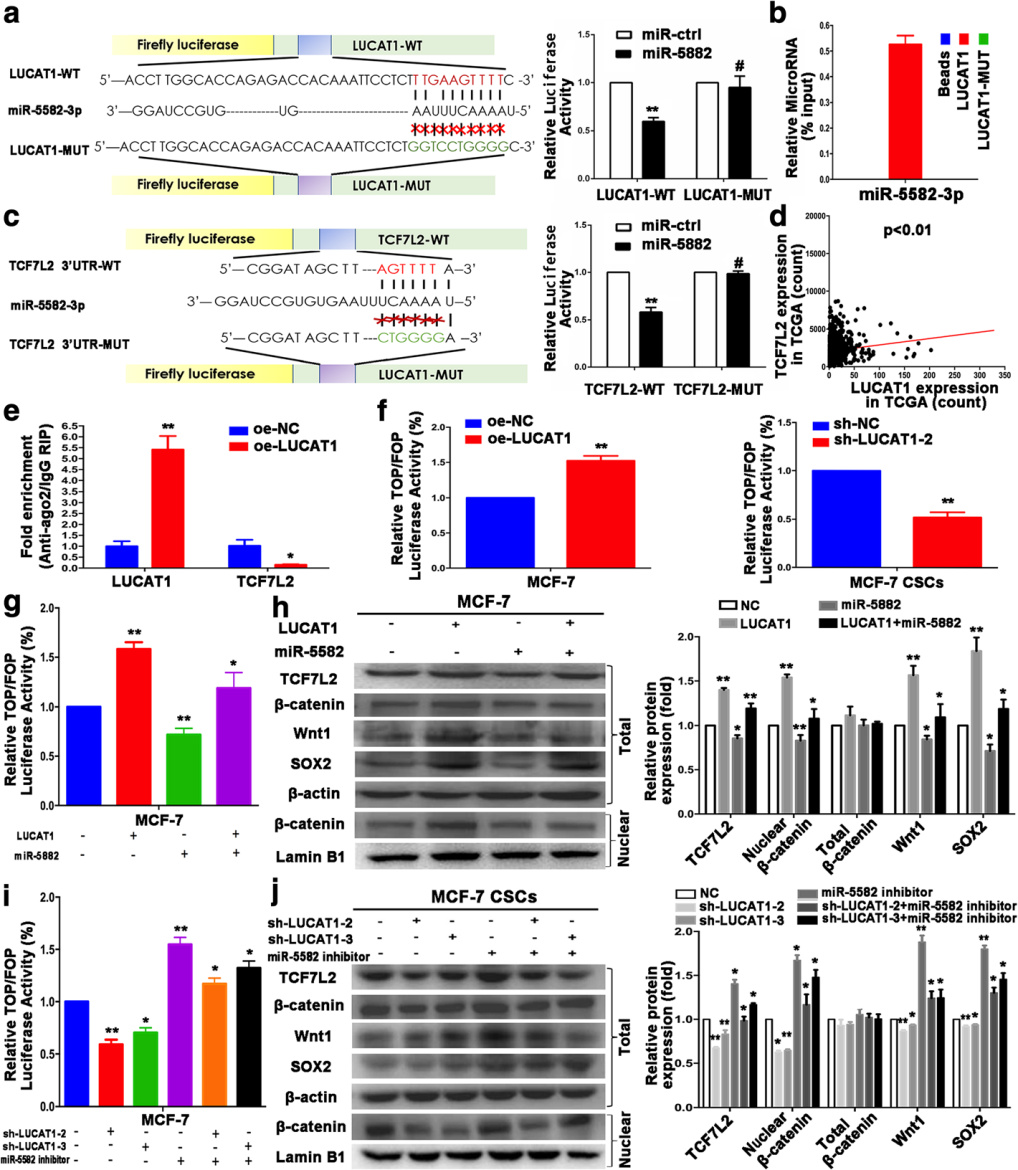
LUCAT1 competitively binds miR-5582-3p with TCF7L2 and enhances Wnt/β-Catenin pathway. **a** Left: The complementary binding of miR-5582-3p and wild/mutant type of LUCAT1. Right: Dual luciferase reporter assays indicated the combination of miR-5582-3p and LUCAT1. **b** MCF-7 cell lysates were incubated with biotinlabeled LUCAT1 and LUCAT1-mut. MiRNA real-time PCR was performed after pull down process. **c** Left: The complementary binding of miR-5582-3p and wild/mutant type of TCF7L2 3′-UTR. Right: Dual luciferase reporter assays showed the combination of miR-5582-3p and TCF7L2 3′-UTR. **d** RNA sequencing data of TCGA database demonstrated that there was a positive correlation between LUCAT1 and TCF7L2 expression. **e** The qRT-PCR results of the RIP based on Ago2 showed that lncRNAs can compete with the TCF7L2 transcript for the binding of miRNAs. **f** The effect on TOP/FOP reporter activity in MCF-7 cells transfected with LUCAT1 overexpression vector or sh-LUCAT1 vector was proved by dual-luciferase assay. A Renilla transfection control normalized all results. **g** The effect on TOP/FOP reporter activity in MCF-7 cells after transfection with control or miR-5582-3p mimic, oe-LUCAT1 or oe-NC plasmid was validated by dual-luciferase assay. **h** SOX2 and Wnt/β-catenin pathway-related protein expression (total TCF7L2 and Wnt1 proteins, total and nuclear β-catenin proteins) were measured by western blot in different groups of MCF-7. **i** The effect on TOP/FOP reporter activity in MCF-7CSCs cells after transfection with control or miR-5582-3p inhibitor, sh-LUCAT1 or sh-NC plasmid, was validated by dual-luciferase assay. **j** SOX2 and Wnt/β-catenin signaling pathway-related protein expression (total TCF7L2 and Wnt1 proteins, total and nuclear β-catenin proteins) were detected by western blot in different groups of MCF-7 CSCs. β-actin and Lamin B1 were used as an internal control and an endogenous control of cell nuclear fraction, respectively. Data are presented as the mean ± SD of three independent experiments performed in triplicate. *P < 0.05, **P < 0.01, ***P < 0.001, ****P < 0.0001

To elucidate whether LUCAT1 functioned as a ceRNA in regulating BC stemness, we used mirDB (http://www.mirdb.org/) and RNA hybrid (https://bibiserv.cebitec.uni-bielefeld.de/rnahybrid/) to predict potential target microRNA of LUCAT1. LUCAT1 might bind to mir-5582-3p, with − 20.2 kcal/mol of binding energy [28] . To confirm the binding function of genes, we performed dual luciferase gene reporter assay. We found miR-5582-3p mimic could inhibit the luciferase activity in LUCAT1-WT group, with no effect in LUCAT1-Mut group (Fig. 5a). Moreover, RNA pull-down assay manifested that miR-5582-3p was more enriched in the wild-type LUCAT1 compared with that in the mutant-type LUCAT1 (Fig. 5b). These results indicated that miR-5582-3p bound to the transcript position of LUCAT1.

To predict the target mRNA of miR-5582-3p, we used RNAhybrid, DIANA (http://diana.imis.athena-innovation.gr/DianaTools/index.php) and Targetscan (http://www.targetscan.org/vert_72/) [29]. MiR-5582-3p might bind to TCF7L2, with − 16.1 kcal/mol of binding energy. TCF7L2 mRNA 3′-UTR including the predicted miR-5582 recognition site (TCF7L2-WT) or the mutation sequence (TCF7L2-MUT) were subcloned into luciferase reporter plasmids. MiR-5582-3p decreased luciferase activity in the WT vector compared with that in MUT type (Fig. 5c). The results confirmed that LUCAT1 was a target gene of miR-5582-3p. We chose mir-5582-3p as a target gene for the following reasons. Firstly, mir-5582-3p has not been reported in breast cancer. Secondly, miR-5582-3p served the opposite function of LUCAT1: (1) High expression in the MCF-7 CSCs and negative correlation with SOX2 expression in tissues. (2) Low expression in LUCAT1-cDNA MCF-7 and high expression in sh-LUCAT1 MCF-7 CSCs. (3) Inhibition of stemness by miR-5582-3p mimic and promotion function by miR-5582-3p inhibitor (Additional file 6: Figure S3a-f).

TCGA indicated a positive correlation between LUCAT1 and TCF7L2 expression (Fig. 5d). In 151 real samples, LUCAT1 mRNA expression (ISH) was positively correlated with TCF7L2 protein expression (IHC) (*p* < 0.001, Table 3). To shed more light on whether LUCAT1 affected the miR-5582/TCF7L2 axis and Wnt pathway, we performed RIP assays based on Ago2, which can enrich for targets bound by miRNAs upon immunoprecipitation [25]. We overexpressed LUCAT1 in MCF-7 cells then pulled down Ago2 using an anti-Ago2 antibody. Overexpression of LUCAT1 caused a significant decrease in the enrichment of TCF7L2 transcripts pulled down by Ago2 (Fig. 5e), indicating that there were less miRNA-bound TCF7L2 transcripts present. This suggested that LUCAT1 can compete with the TCF7L2 transcript for the binding of miRNAs. The transcriptional activity of TOP/FOP was significantly reduced or enhanced in MCF-7 and MCF-7CSCs cells with stable overexpression or down-regulation of LUCAT1 (Fig. 5f). The up-regulation of LUCAT1 in MCF-7 cells could reverse the decline in the transcriptional activity of TOP/FOP by miR-5582-3p mimic (Fig. 5g). The up-regulation of LUCAT1 remarkably increased protein expression of β-catenin in the nucleus, TCF7L2, Wnt1 and SOX2 in total, while miR-5582-3p mimic induced opposite results which could be restored by the co-transfection of oe-LUCAT1 (Fig. 5h). The down-regulation of LUCAT1 expression in MCF-7 CSCs could reverse the enhancement of the transcriptional activity of TOP/FOP by miR-5582-3p inhibitor (Fig. 5i). The similar results of western blot were also observed in MCF-7 CSCs transfected with miR-5582-5p inhibitor and/or sh-LUCAT1 (Fig. 5j). Accumulation and nuclear translocation of β-catenin triggers the activation of the Wnt signaling pathway. Therefore, the β-catenin level was almost unchanged in the total cellular protein fraction. Up- or down-regulation of LUCAT1 obviously modulated the β-catenin expression in the nucleus. The above results indicated that LUCAT1 promoted the accumulation of β-catenin in the nucleus and the activation of classical Wnt pathway. Furthermore, LUCAT1 and miR-5582-3p induced opposite effects on TCF7L2 and Wnt1 expression in mRNA levels (Additional file 7: Figure S4). The LUCAT1/miR-5582-3p/TCF7L2 axis contributed to stem-like properties of BCCs and stemness of BCSCs via Wnt/β-catenin pathway.

**Table 3:** Correlation analysis of LUCAT1 expression and SOX2 and TCF7L2

Factors	Number (%)	LUCAT1 expression		*Spearman rs*	*P value*
		High (%)	Low (%)		
SOX2 expression					
High (%)	81 (53.0)	58 (71.6)	23 (28.4)	0.291	< 0.001
Low (%)	70 (47.0)	30 (42.9)	40 (57.1)		
TCF7L2 expression					
High (%)	76 (53.0)	55 (71.6)	21 (28.4)	0.288	< 0.001
Low (%)	75 (47.0)	33 (42.9)	42 (57.1)		

### LUCAT1 regulates breast cancer stemness in vivo

**Fig. 6:**
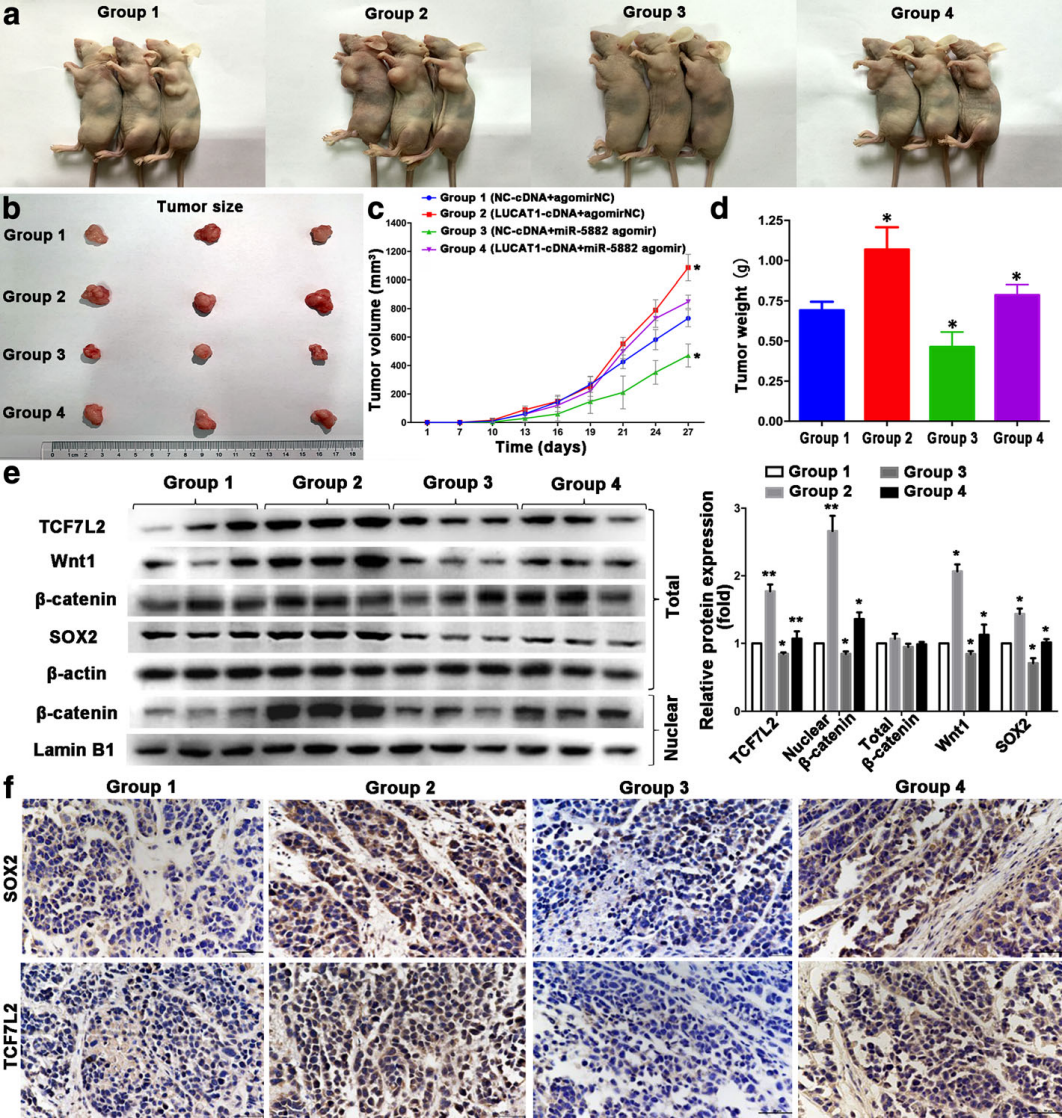
The regulatory mechanism of LUCAT1/miR-5582-3p/TCF7L2 axis and Wnt/β-catenin pathway in vivo. **a** Subcutaneous tumor was taken in four groups. **b** The isolated tumors were separated from xenograft mice. **c** Average tumor volumes were measured in xenograft mice every two days. **d** Images of average tumor weight at the end of indicated treatment. **e** Wnt/β-catenin signaling pathway-related protein expression (total TCF7L2 and Wnt1 proteins, total and nuclear β-catenin proteins) and SOX2 were measured in four groups by western blot. **f** TCF7L2 and SOX2 protein levels were detected in tumor tissues in four groups by IHC. Original magnification, × 400. Scale bars, 50 μm. Data are presented as the mean ± SD of three independent experiments performed in triplicate. *P < 0.05, **P < 0.01, ***P < 0.001, ****P < 0.0001

**Fig. 7:**
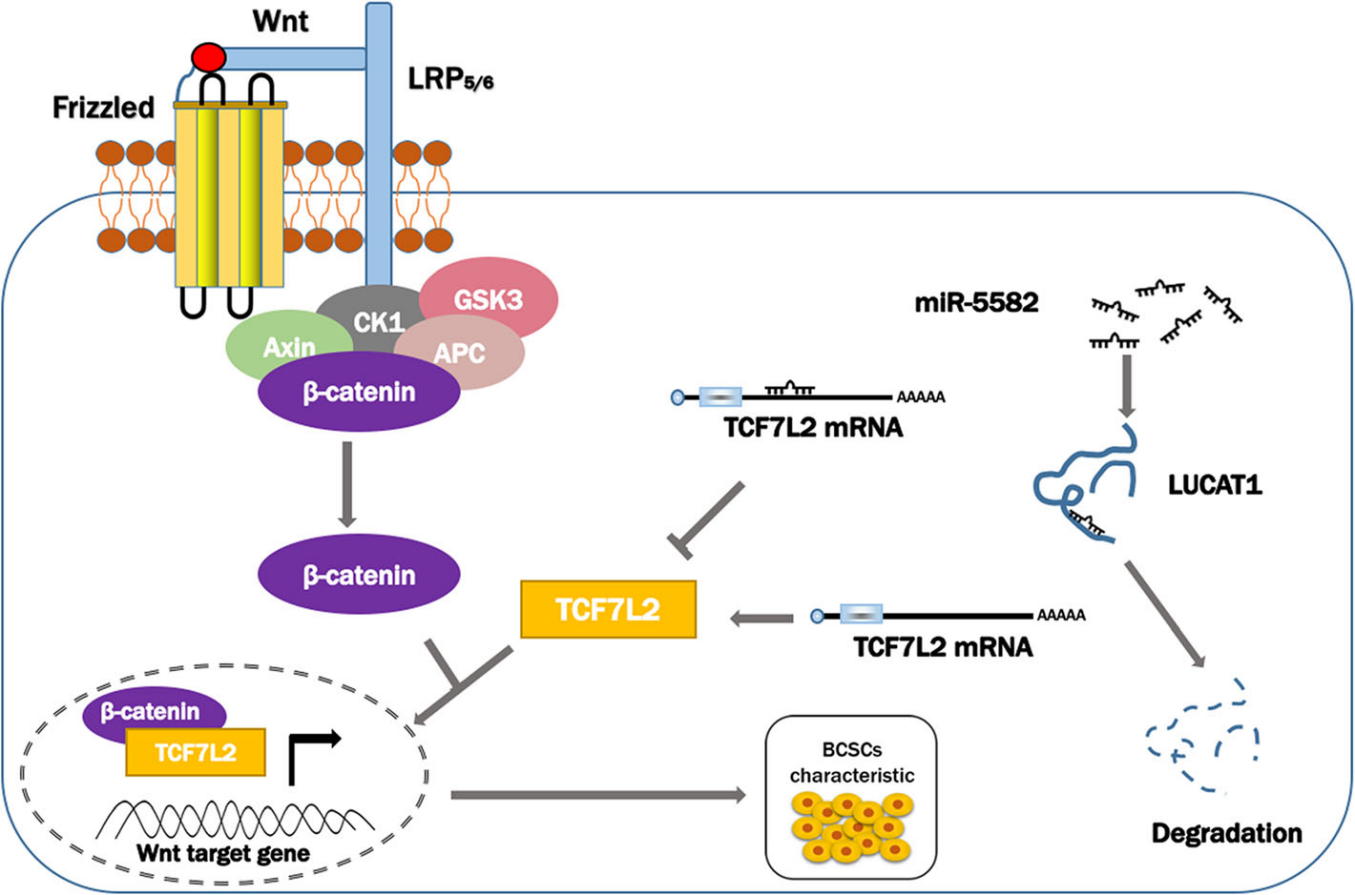
A schematic model showing that LUCAT1 regulates breast cancer stemness by competitively binding to miR-5582-3p and TCF7L2 and consequently regulates the Wnt/β-catenin signaling pathway

To confirm the regulatory mechanism of the whole LUCAT1/miR-5582-3p/TCF7L2 axis and Wnt/β-catenin pathway in vivo, we constructed MCF-7 cell mouse xenograft models (Fig. 6a-b). There were four groups: NC-cDNA mice injected with agomir NC; LUCAT1-cDNA mice injected with agomir NC; NC-cDNA mice injected with miR-5582-3p agomir and LUCAT1-cDNA mice injected with miR-5582-3p agomir. The effect of LUCAT1 on increasing tumor size and weight could be inhibited by miR-5582-3p, while the effect of miR-5582-3p on decreasing tumor size and weight could be rescued when LUCAT1 was overexpressed in the nude mouse model (Fig. 6c-d). Consistent with in vitro findings, the up-regulation of LUCAT1 expression in MCF-7 cells could reverse the decreased expression of β-catenin in nucleus, SOX2, TCF7L2 and Wnt1 in total (Western Blot) in xenograft tumors, caused by miR-5582-3p agomir (Fig. 6e). TCF7L2 and SOX2 expression were also detected by IHC (Fig. 6f). Meanwhile, we investigated the effect of LUCAT1 on tumorigenesis of BCSCs in vivo (Additional file 8: Figure S5a-d). The down-regulation of LUCAT1 expression increased miR-5582-3p expression, and decreased TCF7L2 and Wnt pathway-related proteins expression in MCF-7 CSCs-derived tumors (Additional file 8: Figure S5e-f). All the results verified that LUCAT1 contributed to BC stemness by competitively binding miR-5582-3p with TCF7L2 and enhancing the Wnt/β-catenin signaling pathway (Fig. 7).

## Discussion

Large-scale researches have indicated the essential role of lncRNAs in stemness of CSCs in various cancer. LncRNA XIST promoted glioblastoma CSCs stemness [30]. LncRNA DGCR promoted cancer cell stem-like properties by targeting miR-330-5p/CD44 axis in NSCLC [31]. LncRNA Gata6 increased cancer cell stem-like properties and promoted occurrence in colorectal cancer [32]. LncRNA n339260 promoted CSCs development in hepatocellular carcinoma [33]. Function of lncRNA to CSCs and the pathway to block its regulation is a new task and hotspot in cancer research [34, 35]. Similarly, in breast cancer, new lncRNAs are unceasingly discovered to be involved in regulation of stemness. LncRNA HOTAIR (HOX tran antisense RNA), a 2.2 kilobase ncRNA residing in the *HOXC* locus, indirectly inhibited MiR-7 and contributed to the EMT of BCSCs by activating the STAT3 pathway. LncRNA FEZF1-AS1 regulated BCSCs stemness by sponging miR-30a targeting Nanog [8, 24, 36–38]. In the present study, LUCAT1 had a possible role in promoting BCCs stem-like properties and BCSCs stemness in vitro *and* in vivo*.*

Bioinformatics analysis indicates LUCAT1 may bind with multiple miRNAs. Among them, mir-5582-3p arouses our attention. The precursor of human mir-5582 is located on chromosome 11p11.2, with a length of 68 bp. Previous studies found that mir-5582-3p leaded to apoptosis and cell cycle arrest of tumor cells and its expression in colorectal cancer was significantly lower than that of adjacent normal tissues, with an obvious anti-cancer effect [39]. This study illustrated that mir-5582-3p was a novel suppressor-miR in BCCs stem-like properties and BCSCs stemness via directly targeting the TCF7L2 gene. Further investigations are required to explore the role of mir-5582-3p.

The Wnt/ β-catenin pathway is constitutively active in most tumor cells [40]. It is a recognized pathway in regulating the self-renewal of BCSCs [41]. TCF7L2 is a key effector that formed a TCF7L2/β-catenin complex which transcriptionally activated downstream factors in the Wnt pathway [42]. TCF7L2 represents a central factor in metabolism, stress responses, cell differentiation/proliferation, and cell death [43]. Dysregulation of Wnt/β-catenin pathway and TCF7L2 target genes play a role in carcinogenesis and is especially well documented for breast cancer [44]. The present study indicated that the expression level of nuclear β-catenin and Wnt/β-catenin signaling activity were significantly decreased by the down-regulation of LUCAT1. As the screened lncRNAs participate in many classical pathways such as Hedgehog, Wnt, MAPK and TGF-β pathway, future studies can focus on other signaling pathways in stemness regulation of LUCAT1 [45].

How to convert basic research into clinical practice is a concern to the clinicians. In the tumor cells from breast cancer survivors undergoing chemotherapy, the proportion of BCSCs with CD44^+^CD24^−^ phenotype after chemotherapy was significantly increased compared with that before chemotherapy, and the ability of BCCs to form microspheres was also improved. However, in the tumor cells from Her-2 positive breast cancer patients after treatment with lapatinib, there was no significant increase of CD44^+^CD24^−^ phenotype cells [46]. LUCAT1 and other potential specific therapeutic targets may suppress malignant tumor by inhibiting BCSCs stem phenotype conversion. Traditional chemotherapy drugs and new drugs targeting BCSCs should be combined to reduce the resistance of BCSCs and improve breast cancer treatment.

Our study has some limitations. We selected MCF-7 CSCs and T47D CSCs because changes of stem phenotype in these two cell lines were relatively obvious and the results were stable. More cell lines or primary cell can be used to further verify whether LUCAT1 exerted similar function in cells with various kinds of molecular typing. Although the induction and culture of BCSCs in our research group was approved by previous reports, selection and identification of BCSCs can be improved with more advanced technologies. The effect of BCSCs heterogeneity on the results cannot be neglected.

In-depth study of the molecular regulation mechanism of lncRNAs in BCSCs is expected to enrich the regulation network of gene expression in breast cancer, and explore new diagnostic and specific therapeutic targets for breast cancer.

## Conclusions

LUCAT1 might be a significant biomarker to evaluate prognosis in breast cancer survivors. LUCAT1 increased BCCs stem-like properties and BCSCs stemness by competitively binding miR-5582-3p with TCF7L2 and enhancing the Wnt/β-catenin signaling pathway. The LUCAT1/miR-5582-3p/TCF7L2 axis will provide insights in the mechanisms of stemness regulation and provide theoretical support for finding new diagnostic markers and specific therapeutic targets for breast cancer.

## Additional files

Additional file 1: **Table S1.** The sequences for primers used in the study. (DOCX 17 kb)

Additional file 2: **Table S2.** Antibodies used for IHC and WB. (DOCX 16 kb)

Additional file 3: **Figure S1.** MCF-7 and T47D CSCs were induced and cultured stably. **a** morphology of MCF-7 and T47D changed obviously and size of mammospheres rapidly increased 7–8 days later. **b** The rate of CD44^+^CD24^−^ was detected in MCF-7 and T47D CSCs by flow cytometry. **c** mRNA expression of stemness markers (OCT4, Nanog and SOX2) was detected by qRT-PCR in MCF-7 and T47D CSCs. **d** Protein expression of stemness markers (Nanog, SOX2 and OCT4) was detected in MCF-7 and T47D CSCs by Western blot. Data are presented as the mean ± SD of three independent experiments. *P < 0.05, **P < 0.01, ***P < 0.001, ****P < 0.0001. (DOCX 540 kb)

Additional file 4: **Table S3.** Representative differential expressed lncRNAs in microarray expression profile. (DOCX 16 kb)

Additional file 5: **Figure S2. a** Relative expression of LUCAT1 in cancer and matched adjacent normal tissues were detected in 26 pairs of fresh specimens by qRT-PCR. **b** Correlation of LUCAT1 mRNA expression and SOX2 protein expression was analyzed. **c** LUCAT1 expression was detected in LUCAT1-overexpressing MCF-7 and T47D cells by qRT-PCR. **d** LUCAT1 expression was detected by qRT-PCR in LUCAT1-silencing MCF-7 and T47D CSCs. (DOCX 247 kb)

Additional file 6: **Figure S3. a** Expression of miR-5582-3p was detected in the MCF-7 CSCs and MCF-7 by qRT-PCR. **b** Expression of miR-5582-3p was negatively correlated with SOX2 protein expression in tissues. **c** Expression of miR-5582-3p was detected in the oe-NC and oe-LUCAT1 MCF-7 by qRT-PCR. **d** Expression of miR-5582-3p was detected in sh-NC, sh-LUCAT1–2 and sh-LUCAT1–3 MCF-7 CSCs by qRT-PCR. **e** Stemness markers were detected in MCF-7 which overexpressed miR-5582-3p by qRT-PCR. **f** Stemness markers was detected in MCF-7 CSCs which inhibited miR-5582-3p by Western Blot. (DOCX 324 kb)

Additional file 7: **Figure S4 a** Wnt/β-catenin pathway-related mRNA expression (TCF7L2, Wnt1) was measured after MCF-7 cells transfected with miR-5582-3p inhibitor by qRT-PCR. **b** Wnt/β-catenin pathway-related mRNA expression (TCF7L2, Wnt1) was measured after MCF-7 CSCs transfected with miR-5582-3p mimic by qRT-PCR. **c** Wnt/β-catenin pathway-related mRNA expression (TCF7L2, Wnt1) was measured after MCF-7 cells transfected with LUCAT1-cDNA by qRT-PCR. **d** Wnt/β-catenin pathway-related mRNA expression (TCF7L2, Wnt1) was measured after MCF-7 CSCs transfected with sh-LUCAT1 by qRT-PCR. (DOCX 180 kb)

Additional file 8: **Figure S5 a** Subcutaneous tumor was taken in sh-LUCAT1–2 group and sh-NC group. **b** The isolated tumors were separated from xenograft mice. **c** Average tumor volumes were measured in xenograft mice every two days. **d** Average tumor weight at the end of indicated treatment. **e** TCF7L2 expression were detected in tumor tissues formed from LUCAT1-silencing or NC-silencing MCF-7 CSCs by IHC. Original magnification, × 400. Scale bars, 50 μm. **f** TCF7L2 and Wnt1 in total, β-catenin in the nucleus were measured in sh-NC and sh-LUCAT1 groups by Western Blot. TCF7L2 and miR-5582-3p expression were measured in sh-NC and sh-LUCAT1 groups by qRT-PCR. Data are presented as the mean ± SD of three independent experiments. *P < 0.05, **P < 0.01, ***P < 0.001, ****P < 0.0001. (DOCX 1337 kb)

## Data Availability

All data are fully available without restrictions.

## Abbreviations

AGO2: Argonaute 2
BCCs: Breast cancer cells
BCSCs: Breast cancer stem cells
ceRNA: Competing endogenous RNA
HOTAIR: HOX tran antisense RNA
IHC: Immunohistochemistry
ISH: In situ hybridization
lncRNAs: Long non-coding RNAs
LUCAT1: Lung cancer associated transcript 1
NSCLC: non-small cell lung cancer
qRT-PCR: quantitative real-time polymerase chain reaction
RIP: RNA immunoprecipitation
TCF7L2: Transcription factor 7-like 2)
