# Supplementary material for: Long non-coding RNA LUCAT1/miR-5582-3p/TCF7L2 axis regulates breast cancer stemness via Wnt/β-catenin pathway
Source: J Exp Clin Cancer Res. 2019 Jul 12;38:305. doi: 10.1186/s13046-019-1315-8 (PMC6626338; doi:10.1186/s13046-019-1315-8)
Supplement: Supplementary file 1 — Table S1. The sequences for primers used in the study. (DOCX 17 kb) [file 13046_2019_1315_MOESM1_ESM.docx]

**Additional file 1**

**Table S1.** The sequences for primers used in the study

| **Name** | **Sequences** |
| --- | --- |
| *Primers for qRT-PCR* | |
| U6-F  U6-R | 5’-TGGCACCCAGCACAATGAA-3  5’-CTAAGTCATAGTCCGCCTAGAAGCA-3’ |
| LUCAT1-F | 5’- GCTCGGATTGCCTTAGACAG-3’ |
| LUCAT1-R | 5’- GGGTGAGCTTCTTGTGAGGA-3’ |
| OCT4-F  OCT4-R  C-MYC-F  C-MYC-R | 5′-CTTCGCAAGCCCTCATTTC-3′  5′-GAGAAGGCGAAATCCGAAG-3′  5′-GGCTCCTGGCAAAAGGTCA-3′  5′-CTGCGTAGTTGTGCTGATGT-3′ |
| Nanog-F | 5′-TTTGTGGGCCTGAAGAAAACT-3′ |
| Nanog-R | 5′-AGGGCTGTCCTGAATAAGCAG-3′ |
| SOX2-F | 5′-GCCGAGTGGAAACTTTTGTCG-3′ |
| SOX2-R | 5′-GGCAGCGTGTACTTATCCTTCT-3′ |
| CD44-F | 5′-AAGGTGGAGCAAACACAACC-3′ |
| CD44-R | 5′-TCGACTGTTGACTGCAATGC-3′ |
| TCF7L2-F | 5′-CTCCTCATCAATTGCACAGC-3′ |
| TCF7L2-R | 5′-GGAGCTGTGGGAATGTAACC-3′ |
| Wnt1-F | 5′-ACGTAGCCTCCTCCACGAACCTGC-3′ |
| Wnt1-R | 5′-CGCATCTCGGAGAATACGGTCG-3′ |
| β-actin -F | 5′-CTGGCCGGGACCTGACT-3′ |
| β-actin- R | 5′-TCCTTAATGTCACGCACGATTT -3′ |
| GAPDH-F  GAPDH-R | 5'-GCATCTTCTTGTGCAGTGCC-3'  5'-TACGGCCAAATCCGTTCACA-3' |
| *Sequence of LUCAT1-RNAi* | |
| LUCAT1-RNAi-1 | CCAGACCTCCAGAAACCAT |
| LUCAT1-RNAi-2 | CCTTAGACAGGTGCAATTT |
| LUCAT1-RNAi-3 | GGAACTCTTATGGGACCTT |
| *Target gene mRNA sequence of LUCAT1 probe in ISH*  *Vector system constitution*  LUCAT1-RNAi  LUCAT1-cDNA | 5’ -Dig –ATTGTCACACTATGTGTTCTGACTTCTGGCTCCTTTCCTC–Dig-3’  5’ -Dig –ATGGGCTACATGCTGAGCTACAGAGTTTCGCTCTGTCGCC–Dig-3’  5’ -Dig –CTTAGCGTGCCTGTACAGTTGTGTCCAAATGCTGTCCTCA–Dig-3’  hU6-MCS-Ubiquitin-EGFP-IRES-puromycin  (polyA-MCS-UBI) RV-SV40-EGFP-IRES-puromycin |

Abbreviations: qRT-PCR, Quantitative real-time PCR; ISH, in situ hybridization; F, forward primer; R, reverse primer.
