# Supplementary material for: Long non-coding RNA LUCAT1/miR-5582-3p/TCF7L2 axis regulates breast cancer stemness via Wnt/β-catenin pathway
Source: J Exp Clin Cancer Res. 2019 Jul 12;38:305. doi: 10.1186/s13046-019-1315-8 (PMC6626338; doi:10.1186/s13046-019-1315-8)
Supplement: Supplementary file 2 — Table S2. Antibodies used for IHC and WB. (DOCX 16 kb) [file 13046_2019_1315_MOESM2_ESM.docx]

**Additional file 2**

**Table S2.** Antibodies used for IHC and WB

| **Antibody** | **Company/Provider** | **Dilution ratio** |
| --- | --- | --- |
| anti-human TCF7L2 | Cell Signaling Technology | 1:1000 (WB); 1:200(IHC) |
| anti-human β-catenin | Cell Signaling Technology | 1:1000 |
| anti-human Nanog | Cell Signaling Technology | 1:1000 |
| anti-human OCT4 | Cell Signaling Technology | 1:1000 |
| anti-human SOX2 | Cell Signaling Technology | 1:1000 (WB); 1:200(IHC) |
| anti-human Wnt1 | ABclonal | 1:1000 |
| anti-human β-actin | BOSTER | 1:1000 |
| anti-human Lamin B1 | Cell Signaling Technology | 1:1000 |
| goat anti-mouse IgG | EARTHOX Life Science | 1:10000 |
| goat anti-rabbit IgG | EARTHOX Life Science | 1:10000 |
