# Supplementary material for: Long non-coding RNA LUCAT1/miR-5582-3p/TCF7L2 axis regulates breast cancer stemness via Wnt/β-catenin pathway
Source: J Exp Clin Cancer Res. 2019 Jul 12;38:305. doi: 10.1186/s13046-019-1315-8 (PMC6626338; doi:10.1186/s13046-019-1315-8)
Supplement: Supplementary file 3 — Figure S1. MCF-7 and T47D CSCs were induced and cultured stably. a morphology of MCF-7 and T47D changed obviously and size of mammospheres rapidly increased 7–8 days later. b The rate of CD44+CD24− was detected in MCF-7 and T47D CSCs by flow cytometry. c mRNA expression of stemness markers (OCT4, Nanog and SOX2) was detected by qRT-PCR in MCF-7 and T47D CSCs. d Protein expression of stemness markers (Nanog, SOX2 and OCT4) was detected in MCF-7 and T47D CSCs by Western blot. Data are presented as the mean ± SD of three independent experiments. *P < 0.05, **P < 0.01, ***P < 0.001, ****P < 0.0001. (DOCX 540 kb) [file 13046_2019_1315_MOESM3_ESM.docx]

**Additional file 3: Figure S1**

**
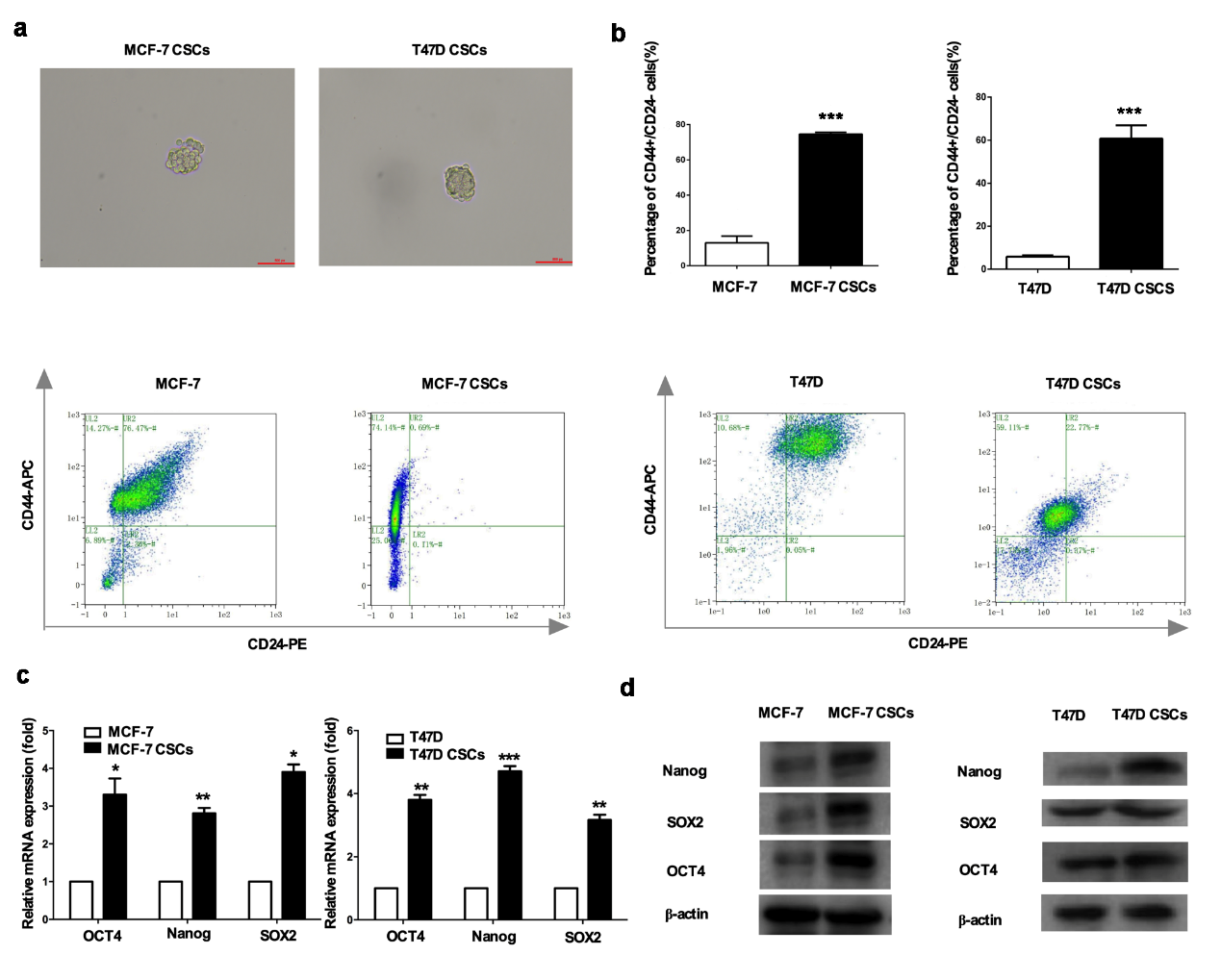
**

**Figure S1**

MCF-7 and T47D CSCs were induced and cultured stably. **a** morphology of MCF-7 and T47D changed obviously and size of mammospheres rapidly increased 7-8 days later. **b** The rate of CD44^+^CD24^−^ was detected in MCF-7 and T47D CSCs by flow cytometry. **c** mRNA expression of stemness markers (OCT4, Nanog and SOX2) was detected by qRT-PCR in MCF-7 and T47D CSCs. **d** Protein expression of stemness markers (Nanog, SOX2 and OCT4) was detected in MCF-7 and T47D CSCs by Western blot. Data are presented as the mean ± SD of three independent experiments. **P* < 0.05, ***P* < 0.01, ****P* < 0.001, *****P* < 0.0001.
