# Supplementary material for: Long non-coding RNA LUCAT1/miR-5582-3p/TCF7L2 axis regulates breast cancer stemness via Wnt/β-catenin pathway
Source: J Exp Clin Cancer Res. 2019 Jul 12;38:305. doi: 10.1186/s13046-019-1315-8 (PMC6626338; doi:10.1186/s13046-019-1315-8)
Supplement: Supplementary file 4 — Table S3. Representative differential expressed lncRNAs in microarray expression profile. (DOCX 16 kb) [file 13046_2019_1315_MOESM4_ESM.docx]

**Additional file 4**

**Table S3.** Representative differential expressed lncRNAs in microarray expression profile

|  | **Up regulated** | **Down regulated** | |
| --- | --- | --- | --- |
| **lncRNA** | **Fold change** | **lncRNA** | **Fold change** |
| LUCAT1 | 5.146 | LINC00565 | -7.02 |
| LINC00887 | 5.120 | LINC01363 | -5.52 |
| XIST | 3.538 | LINC01128 | -4.17 |
| LINC00028 | 3.316 | LINC00308 | -3.64 |
| LINC01011 | 3.249 | LINC01063 | -3.57 |
| LINC01021 | 2.948 | LINC00942 | -3.43 |
| LINC00583 | 2.872 | LINC01000 | -3.30 |
| LINC00240 | 2.642 | LINC00941 | -3.18 |
| LINC00675 | 2.547 | LINC00893 | -3.17 |
| LINC00319 | 2.260 | LINC01184 | -3.04 |
| LINC-ROR | 2.228 | LINC01060 | -2.93 |
| LINC00083 | 2.188 | LINC01089 | -2.82 |
| CASC15 | 2.173 | LINC00662 | -2.81 |
| LINC01018 | 2.153 | LINC00467 | -2.79 |
| LINC01215 | 2.143 | LINC00094 | -2.64 |
| LINC00473 | 2.140 | LINC00999 | -2.61 |
| LINC00484 | 2.128 | LINC00839 | -2.59 |
| LINC00665 | 2.126 | LINC00673 | -2.32 |
