# Supplementary material for: Long non-coding RNA LUCAT1/miR-5582-3p/TCF7L2 axis regulates breast cancer stemness via Wnt/β-catenin pathway
Source: J Exp Clin Cancer Res. 2019 Jul 12;38:305. doi: 10.1186/s13046-019-1315-8 (PMC6626338; doi:10.1186/s13046-019-1315-8)
Supplement: Supplementary file 5 — Figure S2. a Relative expression of LUCAT1 in cancer and matched adjacent normal tissues were detected in 26 pairs of fresh specimens by qRT-PCR. b Correlation of LUCAT1 mRNA expression and SOX2 protein expression was analyzed. c LUCAT1 expression was detected in LUCAT1-overexpressing MCF-7 and T47D cells by qRT-PCR. d LUCAT1 expression was detected by qRT-PCR in LUCAT1-silencing MCF-7 and T47D CSCs. (DOCX 247 kb) [file 13046_2019_1315_MOESM5_ESM.docx]

**Additional file 5: Figure S2**

**
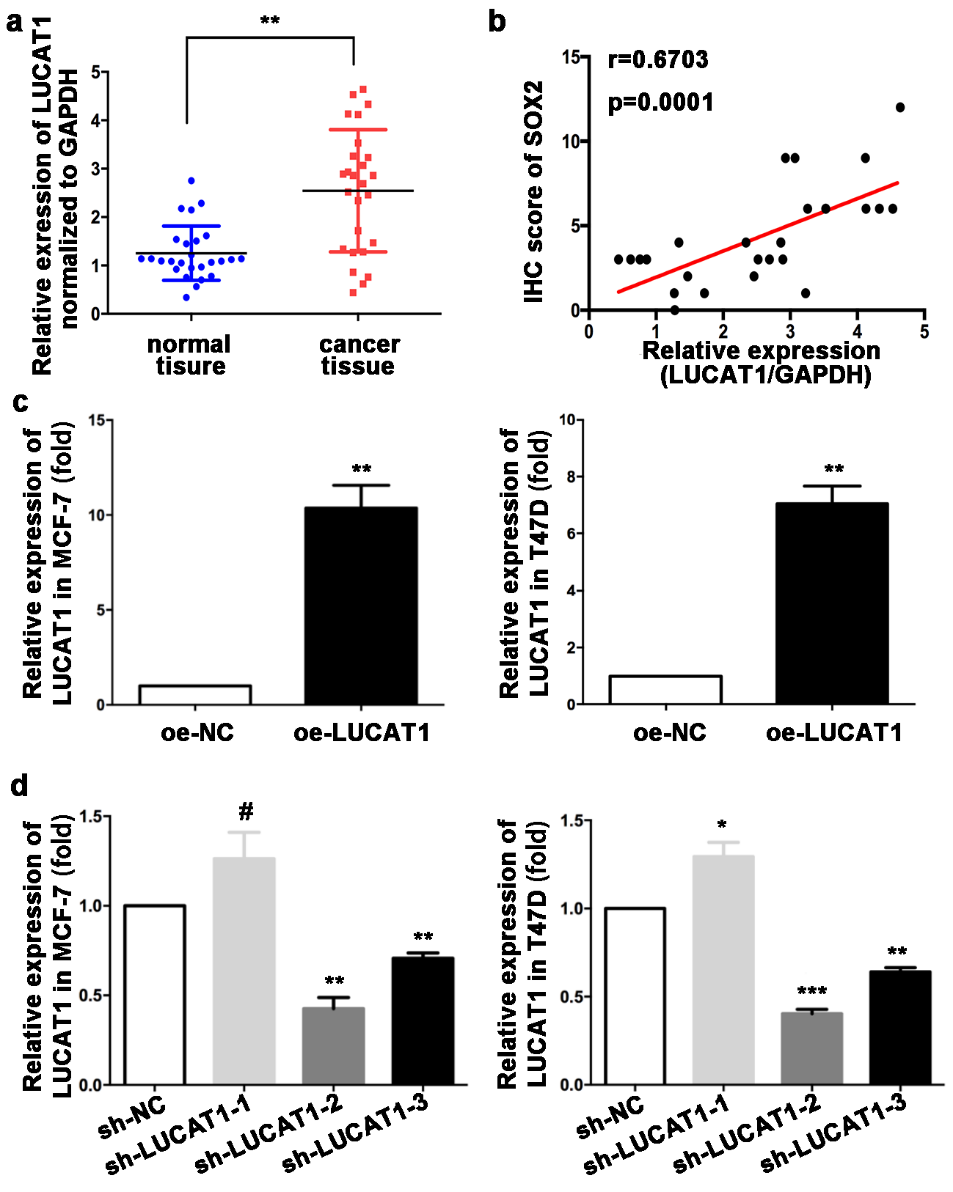
**

**Figure S2**

**a** Relative expression of LUCAT1 in cancer and matched adjacent normal tissues were detected in 26 pairs of fresh specimens by qRT-PCR. **b** Correlation of LUCAT1 mRNA expression and SOX2 protein expression was analyzed. **c** LUCAT1 expression was detected in LUCAT1-overexpressing MCF-7 and T47D cells by qRT-PCR. **d** LUCAT1 expression was detected by qRT-PCR in LUCAT1-silencing MCF-7 and T47D CSCs.
