# Supplementary material for: Long non-coding RNA LUCAT1/miR-5582-3p/TCF7L2 axis regulates breast cancer stemness via Wnt/β-catenin pathway
Source: J Exp Clin Cancer Res. 2019 Jul 12;38:305. doi: 10.1186/s13046-019-1315-8 (PMC6626338; doi:10.1186/s13046-019-1315-8)
Supplement: Supplementary file 6 — Figure S3. a Expression of miR-5582-3p was detected in the MCF-7 CSCs and MCF-7 by qRT-PCR. b Expression of miR-5582-3p was negatively correlated with SOX2 protein expression in tissues. c Expression of miR-5582-3p was detected in the oe-NC and oe-LUCAT1 MCF-7 by qRT-PCR. d Expression of miR-5582-3p was detected in sh-NC, sh-LUCAT1–2 and sh-LUCAT1–3 MCF-7 CSCs by qRT-PCR. e Stemness markers were detected in MCF-7 which overexpressed miR-5582-3p by qRT-PCR. f Stemness markers was detected in MCF-7 CSCs which inhibited miR-5582-3p by Western Blot. (DOCX 324 kb) [file 13046_2019_1315_MOESM6_ESM.docx]

**Additional file 6: Figure S3**


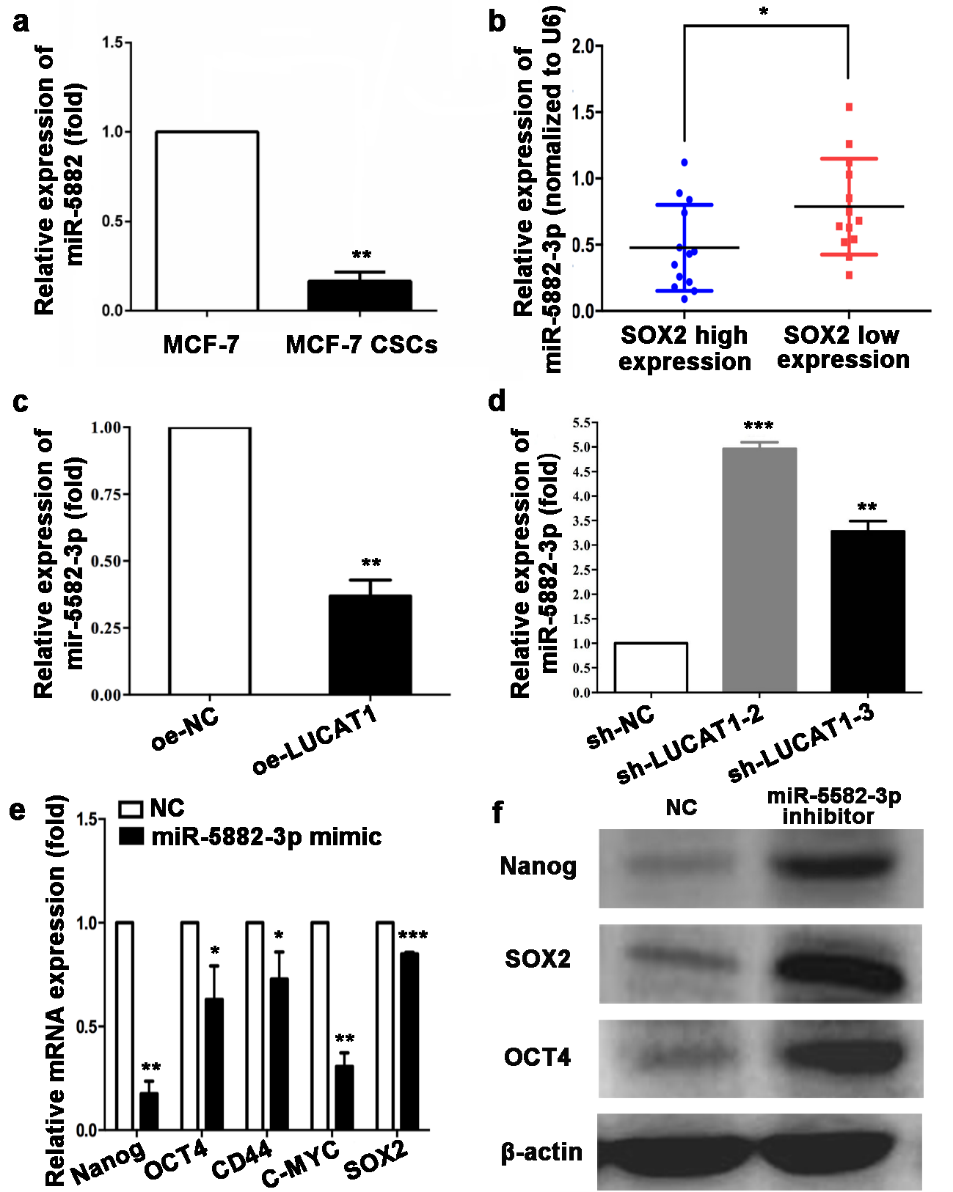


**Figure S3**

**a** Expression of miR-5582-3p was detected in the MCF-7 CSCs and MCF-7 by qRT-PCR. **b** Expression of miR-5582-3p was negatively correlated with SOX2 protein expression in tissues. **c** Expression of miR-5582-3p was detected in the oe-NC and oe-LUCAT1 MCF-7 by qRT-PCR. **d** Expression of miR-5582-3p was detected in sh-NC, sh-LUCAT1-2 and sh-LUCAT1-3 MCF-7 CSCs by qRT-PCR. **e** Stemness markers were detected in MCF-7 which overexpressed miR-5582-3p by qRT-PCR. **f** Stemness markers was detected in MCF-7 CSCs which inhibited miR-5582-3p by Western Blot.
