# Supplementary material for: Long non-coding RNA LUCAT1/miR-5582-3p/TCF7L2 axis regulates breast cancer stemness via Wnt/β-catenin pathway
Source: J Exp Clin Cancer Res. 2019 Jul 12;38:305. doi: 10.1186/s13046-019-1315-8 (PMC6626338; doi:10.1186/s13046-019-1315-8)
Supplement: Supplementary file 7 — Figure S4 a Wnt/β-catenin pathway-related mRNA expression (TCF7L2, Wnt1) was measured after MCF-7 cells transfected with miR-5582-3p inhibitor by qRT-PCR. b Wnt/β-catenin pathway-related mRNA expression (TCF7L2, Wnt1) was measured after MCF-7 CSCs transfected with miR-5582-3p mimic by qRT-PCR. c Wnt/β-catenin pathway-related mRNA expression (TCF7L2, Wnt1) was measured after MCF-7 cells transfected with LUCAT1-cDNA by qRT-PCR. d Wnt/β-catenin pathway-related mRNA expression (TCF7L2, Wnt1) was measured after MCF-7 CSCs transfected with sh-LUCAT1 by qRT-PCR. (DOCX 180 kb) [file 13046_2019_1315_MOESM7_ESM.docx]

**Additional file 7: Figure S4**

**
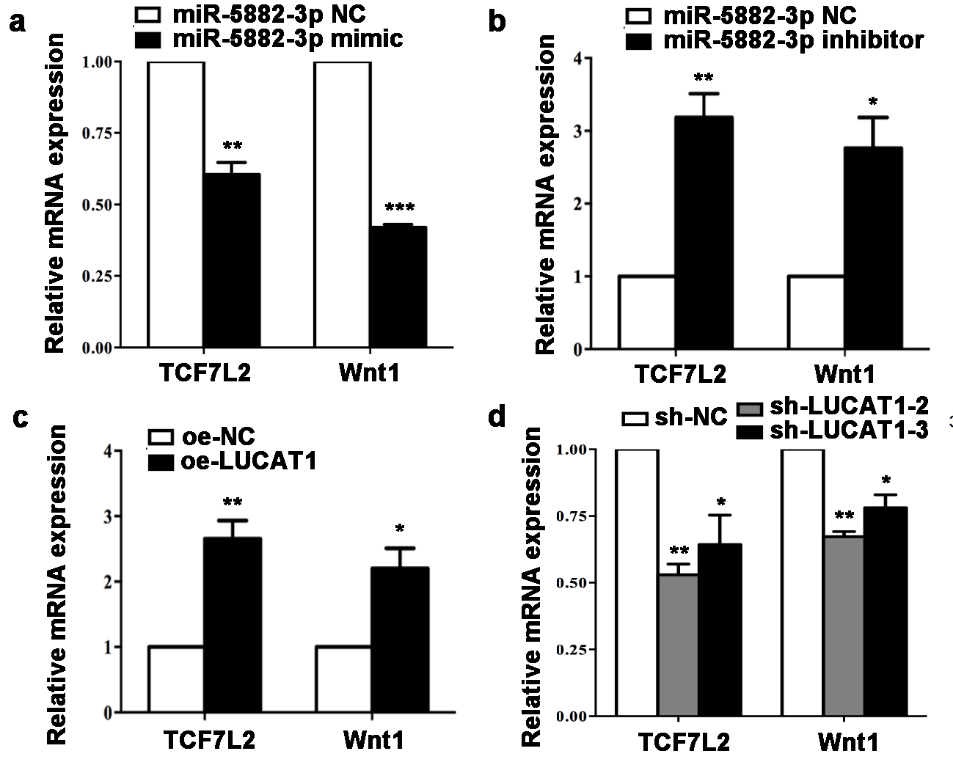
**

**Figure S4**

**a** Wnt/β-catenin pathway-related mRNA expression (TCF7L2, Wnt1) was measured after MCF-7 cells transfected with miR-5582-3p inhibitor by qRT-PCR. **b** Wnt/β-catenin pathway-related mRNA expression (TCF7L2, Wnt1) was measured after MCF-7 CSCs transfected with miR-5582-3p mimic by qRT-PCR. **c** Wnt/β-catenin pathway-related mRNA expression (TCF7L2, Wnt1) was measured after MCF-7 cells transfected with LUCAT1-cDNA by qRT-PCR. **d** Wnt/β-catenin pathway-related mRNA expression (TCF7L2, Wnt1) was measured after MCF-7 CSCs transfected with sh-LUCAT1 by qRT-PCR.
