# Supplementary material for: Long non-coding RNA LUCAT1/miR-5582-3p/TCF7L2 axis regulates breast cancer stemness via Wnt/β-catenin pathway
Source: J Exp Clin Cancer Res. 2019 Jul 12;38:305. doi: 10.1186/s13046-019-1315-8 (PMC6626338; doi:10.1186/s13046-019-1315-8)
Supplement: Supplementary file 8 — Figure S5 a Subcutaneous tumor was taken in sh-LUCAT1–2 group and sh-NC group. b The isolated tumors were separated from xenograft mice. c Average tumor volumes were measured in xenograft mice every two days. d Average tumor weight at the end of indicated treatment. e TCF7L2 expression were detected in tumor tissues formed from LUCAT1-silencing or NC-silencing MCF-7 CSCs by IHC. Original magnification, × 400. Scale bars, 50 μm. f TCF7L2 and Wnt1 in total, β-catenin in the nucleus were measured in sh-NC and sh-LUCAT1 groups by Western Blot. TCF7L2 and miR-5582-3p expression were measured in sh-NC and sh-LUCAT1 groups by qRT-PCR. Data are presented as the mean ± SD of three independent experiments. *P < 0.05, **P < 0.01, ***P < 0.001, ****P < 0.0001. (DOCX 1337 kb) [file 13046_2019_1315_MOESM8_ESM.docx]

**Additional file 8: Figure S5**

**
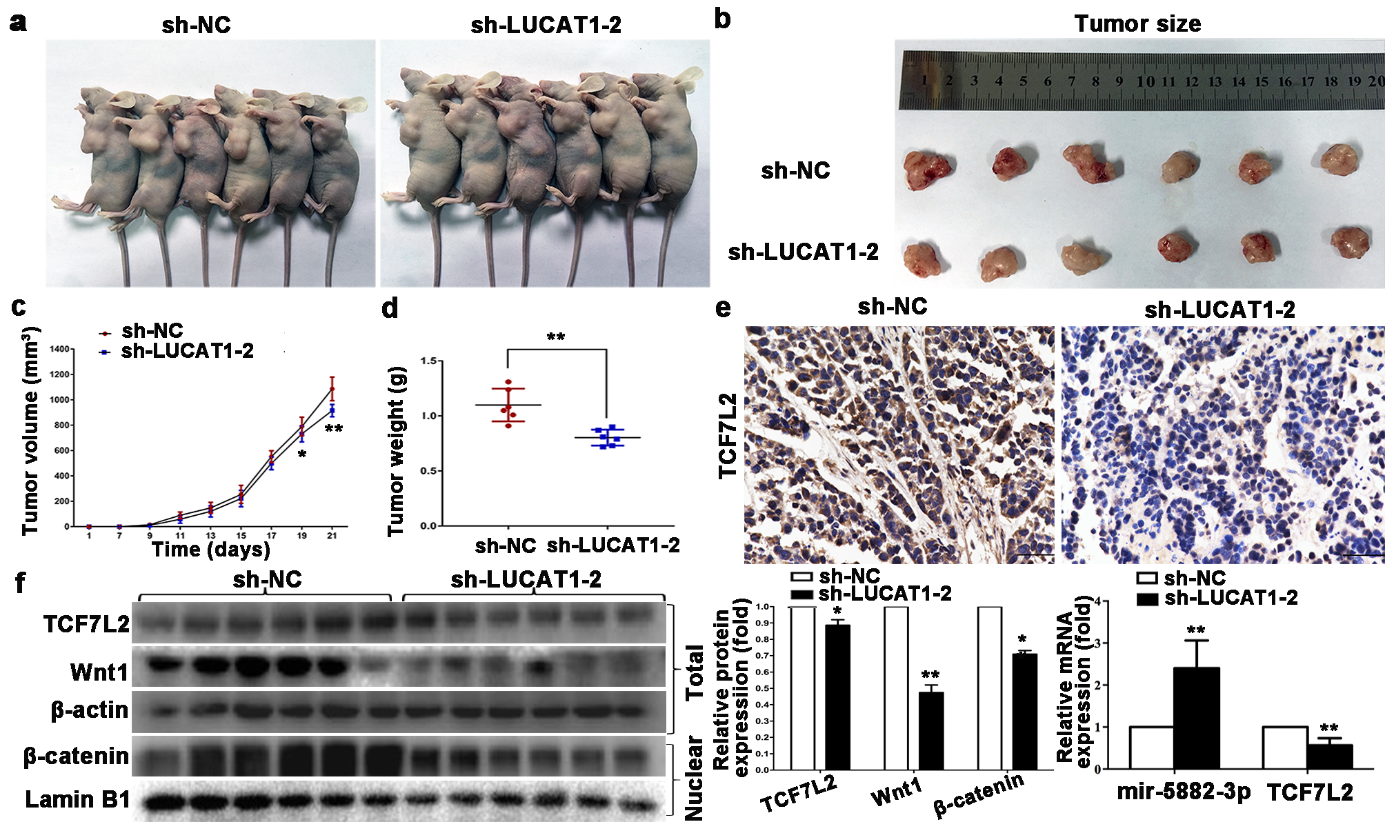
**

**Figure S5**

**a** Subcutaneous tumor was taken in sh-LUCAT1-2 group and sh-NC group. **b** The isolated tumors were separated from xenograft mice. **c** Average tumor volumes were measured in xenograft mice every two days. **d** Average tumor weight at the end of indicated treatment. **e** TCF7L2 expression were detected in tumor tissues formed from LUCAT1-silencing or NC-silencing MCF-7 CSCs by IHC. Original magnification, ×400. Scale bars, 50 μm. **f** TCF7L2 and Wnt1 in total, β-catenin in the nucleus were measured in sh-NC and sh-LUCAT1 groups by Western Blot. TCF7L2 and miR-5582-3p expression were measured in sh-NC and sh-LUCAT1 groups by qRT-PCR. Data are presented as the mean ± SD of three independent experiments. *P < 0.05, **P < 0.01, ***P < 0.001, ****P < 0.0001.
